# Supplementary material for: Ethnicity-Specific Molecular Alterations in MAPK and JAK/STAT Pathways in Early-Onset Colorectal Cancer
Source: Cancers (Basel). 2025 Mar 25;17(7):1093. doi: 10.3390/cancers17071093 (PMC11988162; doi:10.3390/cancers17071093)

Table S1. Alteration rates of JAK/STAT and MAPK pathway-related genes among Early-Onset Colorectal Cancer (EOCRC) and Late-Onset Colorectal Cancer (LOCRC) Hispanic/Latino patients.

Gene	Early Onset H/L n (%)	Late Onset H/L n (%)	p-value
**JAK1 Mutation**			
Present	6 (4.3%)	6 (3.7%)	0.9922
Absent	132 (95.7%)	158 (96.3%)	
**JAK2 Mutation**			
Present	1 (0.7%)	2 (1.2%)	1
Absent	137 (99.3%)	162 (98.8%)	
**JAK3 Mutation**			
Present	4 (2.9%)	6 (3.7%)	0.7591
Absent	134 (97.1%)	158 (96.3%)	
**PIAS1 Mutation**			
Present	0 (0.0%)	0 (0.0%)	1
Absent	138 (100.0%)	164 (100.0%)	
**PIAS2 Mutation**			
Present	0 (0.0%)	0 (0.0%)	1
Absent	138 (100.0%)	164 (100.0%)	
**PIAS3 Mutation**			
Present	0 (0.0%)	0 (0.0%)	1

Absent	138 (100.0%)	164 (100.0%)	
**PTPRC Mutation**			
Present	0 (0.0%)	0 (0.0%)	1
Absent	138 (100.0%)	164 (100.0%)	
**SOCS1 Mutation**			
Present	1 (0.7%)	0 (0.0%)	0.457
Absent	137 (99.3%)	164 (100.0%)	
**STAT1 Mutation**			
Present	0 (0.0%)	0 (0.0%)	1
Absent	138 (100.0%)	164 (100.0%)	
**STAT3 Mutation**			
Present	2 (1.4%)	2 (1.2%)	1
Absent	136 (98.6%)	162 (98.8%)	
**STAT4 Mutation**			
Present	0 (0.0%)	0 (0.0%)	1
Absent	138 (100.0%)	164 (100.0%)	
**STAT5A Mutation**			
Present	2 (1.4%)	6 (3.7%)	0.2973
Absent	136 (98.6%)	158 (96.3%)	
**STAT5B Mutation**			
Present	6 (4.3%)	2 (1.2%)	0.1483
Absent	132 (95.7%)	162 (98.8%)	
**STAT6 Mutation**			
Present	0 (0.0%)	0 (0.0%)	1
Absent	138 (100.0%)	164 (100.0%)	

Gene	Early Onset H/L n (%)	Late Onset H/L n (%)	p-value
**ACVR1 Mutation**			
Present	4 (2.9%)	0 (0.0%)	**0.04257**
Absent	134 (97.1%)	164 (100.0%)	
**ACVR1B Mutation**			
Present	0 (0.0%)	0 (0.0%)	1
Absent	138 (100.0%)	164 (100.0%)	
**ACVR1C Mutation**			
Present	0 (0.0%)	0 (0.0%)	1
Absent	138 (100.0%)	164 (100.0%)	
**AKT1 Mutation**			
Present	7 (5.1%)	3 (1.8%)	0.1948
Absent	131 (94.9%)	161 (98.2%)	
**AKT2 Mutation**			
Present	1 (0.7%)	2 (1.2%)	1
Absent	137 (99.3%)	162 (98.8%)	
**AKT3 Mutation**			
Present	5 (3.6%)	5 (3.0%)	1
Absent	138 (100.0%)	159 (97.0%)	
**ARRB1 Mutation**			
Present	0 (0.0%)	0 (0.0%)	1
Absent	138 (100.0%)	164 (100.0%)	

**ARRB2 Mutation**			
Present	0 (0.0%)	0 (0.0%)	1
Absent	138 (100.0%)	164 (100.0%)	
**ATF2 Mutation**			
Present	0 (0.0%)	0 (0.0%)	1
Absent	138 (100.0%)	164 (100.0%)	
**ATF4 Mutation**			
Present	0 (0.0%)	0 (0.0%)	1
Absent	138 (100.0%)	164 (100.0%)	
**BDNF Mutation**			
Present	0 (0.0%)	0 (0.0%)	1
Absent	138 (100.0%)	164 (100.0%)	
**BRAF Mutation**			
Present	7 (5.1%)	30 (18.3%)	**0.0009188**
Absent	131 (94.9%)	134 (81.7%)	
**CACNA1A Mutation**			
Present	0 (0.0%)	0 (0.0%)	1
Absent	138 (100.0%)	164 (100.0%)	
**CACNA1B Mutation**			
Present	0 (0.0%)	0 (0.0%)	1
Absent	138 (100.0%)	164 (100.0%)	
**CACNA1C Mutation**			
Present	0 (0.0%)	0 (0.0%)	1
Absent	138 (100.0%)	164 (100.0%)	
**CACNA1D Mutation**			

Present	0 (0.0%)	0 (0.0%)	1
Absent	138 (100.0%)	164 (100.0%)	
**CACNA1E Mutation**			
Present	0 (0.0%)	0 (0.0%)	1
Absent	138 (100.0%)	164 (100.0%)	
**CACNAIF Mutation**			
Present	0 (0.0%)	0 (0.0%)	1
Absent	138 (100.0%)	164 (100.0%)	
**CACNA1G Mutation**			
Present	0 (0.0%)	0 (0.0%)	1
Absent	138 (100.0%)	164 (100.0%)	
**CACNA1H Mutation**			
Present	0 (0.0%)	0 (0.0%)	1
Absent	138 (100.0%)	164 (100.0%)	
**CACNA1I Mutation**			
Present	0 (0.0%)	0 (0.0%)	1
Absent	138 (100.0%)	164 (100.0%)	
**CACNA1S Mutation**			
Present	0 (0.0%)	0 (0.0%)	1
Absent	138 (100.0%)	164 (100.0%)	
**CACNA2D1 Mutation**			
Present	0 (0.0%)	0 (0.0%)	1
Absent	138 (100.0%)	164 (100.0%)	
**CACNA2D2 Mutation**			
Present	0 (0.0%)	0 (0.0%)	1

Absent	138 (100.0%)	164 (100.0%)	
**CACNA2D3 Mutation**			
Present	0 (0.0%)	0 (0.0%)	1
Absent	138 (100.0%)	164 (100.0%)	
**CACNA2D4 Mutation**			
Present	0 (0.0%)	0 (0.0%)	1
Absent	138 (100.0%)	164 (100.0%)	
**CACNB1 Mutation**			
Present	0 (0.0%)	0 (0.0%)	1
Absent	138 (100.0%)	164 (100.0%)	
**CACNB2 Mutation**			
Present	0 (0.0%)	0 (0.0%)	1
Absent	138 (100.0%)	164 (100.0%)	
**CACNB3 Mutation**			
Present	0 (0.0%)	0 (0.0%)	1
Absent	138 (100.0%)	164 (100.0%)	
**CACNB4 Mutation**			
Present	0 (0.0%)	0 (0.0%)	1
Absent	138 (100.0%)	164 (100.0%)	
**CACNG1 Mutation**			
Present	0 (0.0%)	0 (0.0%)	1
Absent	138 (100.0%)	164 (100.0%)	
**CACNG2 Mutation**			
Present	0 (0.0%)	0 (0.0%)	1
Absent	138 (100.0%)	164 (100.0%)	

**CACNG3 Mutation**			
Present	0 (0.0%)	0 (0.0%)	1
Absent	138 (100.0%)	164 (100.0%)	
**CACNG4 Mutation**			
Present	0 (0.0%)	0 (0.0%)	1
Absent	138 (100.0%)	164 (100.0%)	
**CACNG5 Mutation**			
Present	0 (0.0%)	0 (0.0%)	1
Absent	138 (100.0%)	164 (100.0%)	
**CACNG6 Mutation**			
Present	0 (0.0%)	0 (0.0%)	1
Absent	138 (100.0%)	164 (100.0%)	
**CACNG7 Mutation**			
Present	0 (0.0%)	0 (0.0%)	1
Absent	138 (100.0%)	164 (100.0%)	
**CACNG8 Mutation**			
Present	0 (0.0%)	0 (0.0%)	1
Absent	138 (100.0%)	164 (100.0%)	
**CASP3 Mutation**			
Present	0 (0.0%)	0 (0.0%)	1
Absent	138 (100.0%)	164 (100.0%)	
**CD14 Mutation**			
Present	0 (0.0%)	0 (0.0%)	1
Absent	138 (100.0%)	164 (100.0%)	
**CDC25B Mutation**			

Present	0 (0.0%)	0 (0.0%)	1
Absent	138 (100.0%)	164 (100.0%)	
**CDC42 Mutation**			
Present	0 (0.0%)	2 (1.2%)	0.5021
Absent	138 (100.0%)	162 (98.8%)	
**CHUK Mutation**			
Present	0 (0.0%)	0 (0.0%)	1
Absent	138 (100.0%)	164 (100.0%)	
**CRK Mutation**			
Present	0 (0.0%)	0 (0.0%)	1
Absent	138 (100.0%)	164 (100.0%)	
**CRKL Mutation**			
Present	0 (0.0%)	0 (0.0%)	1
Absent	138 (100.0%)	164 (100.0%)	
**DAXX Mutation**			
Present	4 (2.9%)	3 (1.8%)	0.7063
Absent	134 (97.1%)	161 (98.2%)	
**DDIT3 Mutation**			
Present	0 (0.0%)	0 (0.0%)	1
Absent	138 (100.0%)	164 (100.0%)	
**DUSP1 Mutation**			
Present	0 (0.0%)	0 (0.0%)	1
Absent	138 (100.0%)	164 (100.0%)	
**DUSP10 Mutation**			
Present	0 (0.0%)	0 (0.0%)	1

Absent	138 (100.0%)	164 (100.0%)	
**DUSP14 Mutation**			
Present	0 (0.0%)	0 (0.0%)	1
Absent	138 (100.0%)	164 (100.0%)	
**DUSP16 Mutation**			
Present	0 (0.0%)	0 (0.0%)	1
Absent	138 (100.0%)	164 (100.0%)	
**DUSP2 Mutation**			
Present	0 (0.0%)	0 (0.0%)	1
Absent	138 (100.0%)	164 (100.0%)	
**DUSP3 Mutation**			
Present	0 (0.0%)	0 (0.0%)	1
Absent	138 (100.0%)	164 (100.0%)	
**DUSP4 Mutation**			
Present	0 (0.0%)	2 (1.2%)	0.5021
Absent	138 (100.0%)	162 (98.8%)	
**DUSP5 Mutation**			
Present	0 (0.0%)	0 (0.0%)	1
Absent	138 (100.0%)	164 (100.0%)	
**DUSP6 Mutation**			
Present	0 (0.0%)	0 (0.0%)	1
Absent	138 (100.0%)	164 (100.0%)	
**DUSP7 Mutation**			
Present	0 (0.0%)	0 (0.0%)	1
Absent	138 (100.0%)	164 (100.0%)	

**DUSP8 Mutation**			
Present	0 (0.0%)	0 (0.0%)	1
Absent	138 (100.0%)	164 (100.0%)	
**DUSP9 Mutation**			
Present	0 (0.0%)	0 (0.0%)	1
Absent	138 (100.0%)	164 (100.0%)	
**ECSIT Mutation**			
Present	0 (0.0%)	0 (0.0%)	1
Absent	138 (100.0%)	164 (100.0%)	
**EGF Mutation**			
Present	0 (0.0%)	0 (0.0%)	1
Absent	138 (100.0%)	164 (100.0%)	
**EGFR Mutation**			
Present	5 (3.6%)	5 (3.0%)	1
Absent	133 (96.4%)	159 (97.0%)	
**ELK1 Mutation**			
Present	0 (0.0%)	0 (0.0%)	1
Absent	138 (100.0%)	164 (100.0%)	
**ELK4 Mutation**			
Present	0 (0.0%)	0 (0.0%)	1
Absent	138 (100.0%)	164 (100.0%)	
**FAS Mutation**			
Present	1 (0.7%)	0 (0.0%)	0.457
Absent	137 (99.3%)	164 (100.0%)	
**FASLG Mutation**			

Present	0 (0.0%)	0 (0.0%)	1
Absent	138 (100.0%)	164 (100.0%)	
**FGF1 Mutation**			
Present	0 (0.0%)	0 (0.0%)	1
Absent	138 (100.0%)	164 (100.0%)	
**FGF10 Mutation**			
Present	0 (0.0%)	0 (0.0%)	1
Absent	138 (100.0%)	164 (100.0%)	
**FGF11 Mutation**			
Present	0 (0.0%)	0 (0.0%)	1
Absent	138 (100.0%)	164 (100.0%)	
**FGF12 Mutation**			
Present	0 (0.0%)	0 (0.0%)	1
Absent	138 (100.0%)	164 (100.0%)	
**FGF13 Mutation**			
Present	0 (0.0%)	0 (0.0%)	1
Absent	138 (100.0%)	164 (100.0%)	
**FGF14 Mutation**			
Present	0 (0.0%)	0 (0.0%)	1
Absent	138 (100.0%)	164 (100.0%)	
**FGF16 Mutation**			
Present	0 (0.0%)	0 (0.0%)	1
Absent	138 (100.0%)	164 (100.0%)	
**FGF17 Mutation**			
Present	0 (0.0%)	0 (0.0%)	1

Absent	138 (100.0%)	164 (100.0%)	
**FGF18 Mutation**			
Present	0 (0.0%)	0 (0.0%)	1
Absent	138 (100.0%)	164 (100.0%)	
**FGF19 Mutation**			
Present	1 (0.7%)	4 (2.4%)	0.3802
Absent	137 (99.3%)	160 (97.6%)	
**FGF2 Mutation**			
Present	0 (0.0%)	0 (0.0%)	1
Absent	138 (100.0%)	164 (100.0%)	
**FGF20 Mutation**			
Present	0 (0.0%)	0 (0.0%)	1
Absent	138 (100.0%)	164 (100.0%)	
**FGF21 Mutation**			
Present	0 (0.0%)	0 (0.0%)	1
Absent	138 (100.0%)	164 (100.0%)	
**FGF22 Mutation**			
Present	0 (0.0%)	0 (0.0%)	1
Absent	138 (100.0%)	164 (100.0%)	
**FGF23 Mutation**			
Present	0 (0.0%)	0 (0.0%)	1
Absent	138 (100.0%)	164 (100.0%)	
**FGF3 Mutation**			
Present	2 (1.4%)	2 (1.2%)	1
Absent	136 (98.6%)	162 (98.8%)	

**FGF4 Mutation**			
Present	0 (0.0%)	1 (0.6%)	1
Absent	138 (100.0%)	163 (99.4%)	
**FGF5 Mutation**			
Present	0 (0.0%)	0 (0.0%)	1
Absent	138 (100.0%)	164 (100.0%)	
**FGF6 Mutation**			
Present	0 (0.0%)	0 (0.0%)	1
Absent	138 (100.0%)	164 (100.0%)	
**FGF7 Mutation**			
Present	0 (0.0%)	0 (0.0%)	1
Absent	138 (100.0%)	164 (100.0%)	
**FGF8 Mutation**			
Present	0 (0.0%)	0 (0.0%)	1
Absent	138 (100.0%)	164 (100.0%)	
**FGF9 Mutation**			
Present	0 (0.0%)	0 (0.0%)	1
Absent	138 (100.0%)	164 (100.0%)	
**FGFR1 Mutation**			
Present	2 (1.4%)	4 (2.4%)	0.6914
Absent	136 (98.6%)	160 (97.6%)	
**FGFR2 Mutation**			
Present	5 (3.6%)	2 (1.2%)	0.2526
Absent	133 (96.4%)	162 (98.8%)	
**FGFR3 Mutation**			

Present	5 (3.6%)	7 (4.3%)	1
Absent	133 (96.4%)	157 (95.7%)	
**FGFR4 Mutation**			
Present	3 (2.2%)	6 (3.7%)	0.5161
Absent	135 (97.8%)	158 (96.3%)	
**FLNA Mutation**			
Present	0 (0.0%)	0 (0.0%)	1
Absent	138 (100.0%)	164 (100.0%)	
**FLNB Mutation**			
Present	0 (0.0%)	0 (0.0%)	1
Absent	138 (100.0%)	164 (100.0%)	
**FLNC Mutation**			
Present	0 (0.0%)	0 (0.0%)	1
Absent	138 (100.0%)	164 (100.0%)	
**FOS Mutation**			
Present	0 (0.0%)	0 (0.0%)	1
Absent	138 (100.0%)	164 (100.0%)	
**GADD45A Mutation**			
Present	0 (0.0%)	0 (0.0%)	1
Absent	138 (100.0%)	164 (100.0%)	
**GADD45B Mutation**			
Present	0 (0.0%)	0 (0.0%)	1
Absent	138 (100.0%)	164 (100.0%)	
**GADD45G Mutation**			
Present	0 (0.0%)	0 (0.0%)	1

Absent	138 (100.0%)	164 (100.0%)	
**GNA12 Mutation**			
Present	0 (0.0%)	0 (0.0%)	1
Absent	138 (100.0%)	164 (100.0%)	
**GNG12 Mutation**			
Present	0 (0.0%)	0 (0.0%)	1
Absent	138 (100.0%)	164 (100.0%)	
**GRB2 Mutation**			
Present	0 (0.0%)	0 (0.0%)	1
Absent	138 (100.0%)	164 (100.0%)	
**HRAS Mutation**			
Present	2 (1.4%)	4 (2.4%)	0.6914
Absent	136 (98.6%)	160 (97.6%)	
**IKBKB Mutation**			
Present	0 (0.0%)	0 (0.0%)	1
Absent	138 (100.0%)	164 (100.0%)	
**IKBKG Mutation**			
Present	0 (0.0%)	0 (0.0%)	1
Absent	138 (100.0%)	164 (100.0%)	
**IL1A Mutation**			
Present	0 (0.0%)	0 (0.0%)	1
Absent	138 (100.0%)	164 (100.0%)	
**IL1B Mutation**			
Present	0 (0.0%)	0 (0.0%)	1
Absent	138 (100.0%)	164 (100.0%)	

**IL1R1 Mutation**			
Present	0 (0.0%)	0 (0.0%)	1
Absent	138 (100.0%)	164 (100.0%)	
**IL1R2 Mutation**			
Present	0 (0.0%)	0 (0.0%)	1
Absent	138 (100.0%)	164 (100.0%)	
**JUN Mutation**			
Present	3 (2.2%)	1 (0.6%)	0.3349
Absent	135 (97.8%)	163 (99.4%)	
**JUND Mutation**			
Present	0 (0.0%)	0 (0.0%)	1
Absent	138 (100.0%)	164 (100.0%)	
**KRAS Mutation**			
Present	51 (37.0%)	68 (41.5%)	0.4964
Absent	86 (62.3%)	95 (57.9%)	
**MAP2K1 Mutation**			
Present	5 (3.6%)	0 (0.0%)	**0.01914**
Absent	133 (96.4%)	164 (100.0%)	
**MAP2K2 Mutation**			
Present	3 (2.2%)	5 (3.0%)	0.7312
Absent	135 (97.8%)	159 (97.0%)	
**MAP2K3 Mutation**			
Present	0 (0.0%)	0 (0.0%)	1
Absent	138 (100.0%)	164 (100.0%)	
**MAP2K4 Mutation**			

Present	6 (4.3%)	4 (2.4%)	0.5209
Absent	132 (95.7%)	160 (97.6%)	
**MAP2K5 Mutation**			
Present	0 (0.0%)	0 (0.0%)	1
Absent	138 (100.0%)	164 (100.0%)	
**MAP2K6 Mutation**			
Present	0 (0.0%)	0 (0.0%)	1
Absent	138 (100.0%)	164 (100.0%)	
**MAP2K7 Mutation**			
Present	0 (0.0%)	0 (0.0%)	1
Absent	138 (100.0%)	164 (100.0%)	
**MAP3K1 Mutation**			
Present	7 (5.1%)	3 (1.8%)	0.1948
Absent	131 (94.9%)	161 (98.2%)	
**MAP3K10 Mutation**			
Present	0 (0.0%)	0 (0.0%)	1
Absent	138 (100.0%)	164 (100.0%)	
**MAP3K12 Mutation**			
Present	0 (0.0%)	0 (0.0%)	1
Absent	138 (100.0%)	164 (100.0%)	
**MAP3K13 Mutation**			
Present	2 (1.4%)	3 (1.8%)	1
Absent	136 (98.6%)	161 (98.2%)	
**MAP3K14 Mutation**			
Present	0 (0.0%)	0 (0.0%)	1

Absent	138 (100.0%)	164 (100.0%)	
**MAP3K2 Mutation**			
Present	0 (0.0%)	0 (0.0%)	1
Absent	138 (100.0%)	164 (100.0%)	
**MAP3K3 Mutation**			
Present	0 (0.0%)	0 (0.0%)	1
Absent	138 (100.0%)	164 (100.0%)	
**MAP3K4 Mutation**			
Present	0 (0.0%)	0 (0.0%)	1
Absent	138 (100.0%)	164 (100.0%)	
**MAP3K5 Mutation**			
Present	0 (0.0%)	0 (0.0%)	1
Absent	138 (100.0%)	164 (100.0%)	
**MAP3K6 Mutation**			
Present	1 (0.7%)	0 (0.0%)	0.457
Absent	137 (99.3%)	164 (100.0%)	
**MAP3K7 Mutation**			
Present	0 (0.0%)	0 (0.0%)	1
Absent	138 (100.0%)	164 (100.0%)	
**MAP3K8 Mutation**			
Present	0 (0.0%)	0 (0.0%)	1
Absent	138 (100.0%)	164 (100.0%)	
**MAP4K1 Mutation**			
Present	0 (0.0%)	0 (0.0%)	1
Absent	138 (100.0%)	164 (100.0%)	

**MAP4K2 Mutation**			
Present	0 (0.0%)	0 (0.0%)	1
Absent	138 (100.0%)	164 (100.0%)	
**MAP4K3 Mutation**			
Present	0 (0.0%)	0 (0.0%)	1
Absent	138 (100.0%)	164 (100.0%)	
**MAP4K4 Mutation**			
Present	1 (0.7%)	0 (0.0%)	0.457
Absent	137 (99.3%)	164 (100.0%)	
**MAPK1 Mutation**			
Present	2 (1.4%)	0 (0.0%)	0.208
Absent	136 (98.6%)	164 (100.0%)	
**MAPK10 Mutation**			
Present	0 (0.0%)	0 (0.0%)	1
Absent	138 (100.0%)	164 (100.0%)	
**MAPK11 Mutation**			
Present	0 (0.0%)	0 (0.0%)	1
Absent	138 (100.0%)	164 (100.0%)	
**MAPK12 Mutation**			
Present	0 (0.0%)	0 (0.0%)	1
Absent	138 (100.0%)	164 (100.0%)	
**MAPK13 Mutation**			
Present	0 (0.0%)	0 (0.0%)	1
Absent	138 (100.0%)	164 (100.0%)	
**MAPK14 Mutation**			

Present	0 (0.0%)	0 (0.0%)	1
Absent	138 (100.0%)	164 (100.0%)	
**MAPK3 Mutation**			
Present	5 (3.6%)	1 (0.6%)	0.09657
Absent	133 (96.4%)	163 (99.4%)	
**MAPK7 Mutation**			
Present	0 (0.0%)	0 (0.0%)	1
Absent	138 (100.0%)	164 (100.0%)	
**MAPK8 Mutation**			
Present	0 (0.0%)	0 (0.0%)	1
Absent	138 (100.0%)	164 (100.0%)	
**MAPK8IP1 Mutation**			
Present	0 (0.0%)	0 (0.0%)	1
Absent	138 (100.0%)	164 (100.0%)	
**MAPK8IP2 Mutation**			
Present	0 (0.0%)	0 (0.0%)	1
Absent	138 (100.0%)	164 (100.0%)	
**MAPK8IP3 Mutation**			
Present	0 (0.0%)	0 (0.0%)	1
Absent	138 (100.0%)	164 (100.0%)	
**MAPK9 Mutation**			
Present	0 (0.0%)	0 (0.0%)	1
Absent	138 (100.0%)	164 (100.0%)	
**MAPKAPK2 Mutation**			
Present	0 (0.0%)	0 (0.0%)	1

Absent	138 (100.0%)	164 (100.0%)	
**MAPKAPK3 Mutation**			
Present	0 (0.0%)	0 (0.0%)	1
Absent	138 (100.0%)	164 (100.0%)	
**MAPKAPK5 Mutation**			
Present	0 (0.0%)	0 (0.0%)	1
Absent	138 (100.0%)	164 (100.0%)	
**MAPT Mutation**			
Present	0 (0.0%)	0 (0.0%)	1
Absent	138 (100.0%)	164 (100.0%)	
**MAX Mutation**			
Present	0 (0.0%)	0 (0.0%)	1
Absent	138 (100.0%)	164 (100.0%)	
**MEF2C Mutation**			
Present	0 (0.0%)	0 (0.0%)	1
Absent	138 (100.0%)	164 (100.0%)	
**MKNK1 Mutation**			
Present	0 (0.0%)	0 (0.0%)	1
Absent	138 (100.0%)	164 (100.0%)	
**MKNK2 Mutation**			
Present	0 (0.0%)	0 (0.0%)	1
Absent	138 (100.0%)	164 (100.0%)	
**MOS Mutation**			
Present	0 (0.0%)	0 (0.0%)	1
Absent	138 (100.0%)	164 (100.0%)	

**MRAS Mutation**			
Present	0 (0.0%)	0 (0.0%)	1
Absent	138 (100.0%)	164 (100.0%)	
**MYC Mutation**			
Present	1 (0.7%)	2 (1.2%)	1
Absent	137 (99.3%)	162 (98.8%)	
**NF1 Mutation**			
Present	16 (11.6%)	6 (3.7%)	**0.01547**
Absent	122 (88.4%)	158 (96.3%)	
**NFATC2 Mutation**			
Present	0 (0.0%)	0 (0.0%)	1
Absent	138 (100.0%)	164 (100.0%)	
**NFATC4 Mutation**			
Present	0 (0.0%)	0 (0.0%)	1
Absent	138 (100.0%)	164 (100.0%)	
**NFKB1 Mutation**			
Present	0 (0.0%)	0 (0.0%)	1
Absent	138 (100.0%)	164 (100.0%)	
**NFKB2 Mutation**			
Present	0 (0.0%)	0 (0.0%)	1
Absent	138 (100.0%)	164 (100.0%)	
**NLK Mutation**			
Present	0 (0.0%)	0 (0.0%)	1
Absent	138 (100.0%)	164 (100.0%)	
**NR4A1 Mutation**			

Present	0 (0.0%)	0 (0.0%)	1
Absent	138 (100.0%)	164 (100.0%)	
**NRAS Mutation**			
Present	8 (5.8%)	9 (5.5%)	1
Absent	130 (94.2%)	155 (94.5%)	
**NTF3 Mutation**			
Present	0 (0.0%)	0 (0.0%)	1
Absent	138 (100.0%)	164 (100.0%)	
**NTRK1 Mutation**			
Present	5 (3.6%)	5 (3.0%)	1
Absent	133 (96.4%)	159 (97.0%)	
**NTRK2 Mutation**			
Present	4 (2.9%)	6 (3.7%)	0.7591
Absent	134 (97.1%)	158 (96.3%)	
**PAK1 Mutation**			
Present	3 (2.2%)	0 (0.0%)	0.09429
Absent	135 (97.8%)	164 (100.0%)	
**PAK2 Mutation**			
Present	0 (0.0%)	0 (0.0%)	1
Absent	138 (100.0%)	164 (100.0%)	
**PDGFA Mutation**			
Present	0 (0.0%)	0 (0.0%)	1
Absent	138 (100.0%)	164 (100.0%)	
**PDGFB Mutation**			
Present	0 (0.0%)	0 (0.0%)	1

Absent	138 (100.0%)	164 (100.0%)	
**PDGFRA Mutation**			
Present	8 (5.8%)	3 (1.8%)	0.1196
Absent	130 (94.2%)	161 (98.2%)	
**PDGFRB Mutation**			
Present	8 (5.8%)	3 (1.8%)	0.1196
Absent	130 (94.2%)	161 (98.2%)	
**PLA2G10 Mutation**			
Present	0 (0.0%)	0 (0.0%)	1
Absent	138 (100.0%)	164 (100.0%)	
**PLA2G12A Mutation**			
Present	0 (0.0%)	0 (0.0%)	1
Absent	138 (100.0%)	164 (100.0%)	
**PLA2G12B Mutation**			
Present	0 (0.0%)	0 (0.0%)	1
Absent	138 (100.0%)	164 (100.0%)	
**PLA2G1B Mutation**			
Present	0 (0.0%)	0 (0.0%)	1
Absent	138 (100.0%)	164 (100.0%)	
**PLA2G2A Mutation**			
Present	0 (0.0%)	0 (0.0%)	1
Absent	138 (100.0%)	164 (100.0%)	
**PLA2G2D Mutation**			
Present	0 (0.0%)	0 (0.0%)	1
Absent	138 (100.0%)	164 (100.0%)	

**PLA2G2E Mutation**			
Present	0 (0.0%)	0 (0.0%)	1
Absent	138 (100.0%)	164 (100.0%)	
**PLA2G2F Mutation**			
Present	0 (0.0%)	0 (0.0%)	1
Absent	138 (100.0%)	164 (100.0%)	
**PLA2G3 Mutation**			
Present	0 (0.0%)	0 (0.0%)	1
Absent	138 (100.0%)	164 (100.0%)	
**PLA2G4A Mutation**			
Present	0 (0.0%)	0 (0.0%)	1
Absent	138 (100.0%)	164 (100.0%)	
**PLA2G5 Mutation**			
Present	0 (0.0%)	0 (0.0%)	1
Absent	138 (100.0%)	164 (100.0%)	
**PLA2G6 Mutation**			
Present	0 (0.0%)	0 (0.0%)	1
Absent	138 (100.0%)	164 (100.0%)	
**PPM1A Mutation**			
Present	0 (0.0%)	0 (0.0%)	1
Absent	138 (100.0%)	164 (100.0%)	
**PPM1B Mutation**			
Present	0 (0.0%)	0 (0.0%)	1
Absent	138 (100.0%)	164 (100.0%)	
**PPP3CA Mutation**			

Present	0 (0.0%)	0 (0.0%)	1
Absent	138 (100.0%)	164 (100.0%)	
**PPP3CB Mutation**			
Present	0 (0.0%)	0 (0.0%)	1
Absent	138 (100.0%)	164 (100.0%)	
**PPP3CC Mutation**			
Present	0 (0.0%)	0 (0.0%)	1
Absent	138 (100.0%)	164 (100.0%)	
**PPP3R1 Mutation**			
Present	0 (0.0%)	0 (0.0%)	1
Absent	138 (100.0%)	164 (100.0%)	
**PPP3R2 Mutation**			
Present	0 (0.0%)	0 (0.0%)	1
Absent	138 (100.0%)	164 (100.0%)	
**PPP5C Mutation**			
Present	0 (0.0%)	0 (0.0%)	1
Absent	138 (100.0%)	164 (100.0%)	
**PRKACA Mutation**			
Present	0 (0.0%)	0 (0.0%)	1
Absent	138 (100.0%)	164 (100.0%)	
**PRKACB Mutation**			
Present	0 (0.0%)	0 (0.0%)	1
Absent	138 (100.0%)	164 (100.0%)	
**PRKACG Mutation**			
Present	0 (0.0%)	0 (0.0%)	1

Absent	138 (100.0%)	164 (100.0%)	
**PRKCA Mutation**			
Present	0 (0.0%)	0 (0.0%)	1
Absent	138 (100.0%)	164 (100.0%)	
**PRKCG Mutation**			
Present	0 (0.0%)	0 (0.0%)	1
Absent	138 (100.0%)	164 (100.0%)	
**PRKX Mutation**			
Present	0 (0.0%)	0 (0.0%)	1
Absent	138 (100.0%)	164 (100.0%)	
**PRKY Mutation**			
Present	0 (0.0%)	0 (0.0%)	1
Absent	138 (100.0%)	164 (100.0%)	
**PTPN5 Mutation**			
Present	0 (0.0%)	0 (0.0%)	1
Absent	138 (100.0%)	164 (100.0%)	
**PTPN7 Mutation**			
Present	0 (0.0%)	0 (0.0%)	1
Absent	138 (100.0%)	164 (100.0%)	
**PTPRR Mutation**			
Present	0 (0.0%)	0 (0.0%)	1
Absent	138 (100.0%)	164 (100.0%)	
**RAC1 Mutation**			
Present	1 (0.7%)	0 (0.0%)	0.457
Absent	137 (99.3%)	164 (100.0%)	

**RAC2 Mutation**			
Present	1 (0.7%)	1 (0.6%)	1
Absent	137 (99.3%)	163 (99.4%)	
**RAC3 Mutation**			
Present	0 (0.0%)	0 (0.0%)	1
Absent	138 (100.0%)	164 (100.0%)	
**RAF1 Mutation**			
Present	3 (2.2%)	3 (1.8%)	1
Absent	135 (97.8%)	161 (98.2%)	
**RAP1A Mutation**			
Present	0 (0.0%)	0 (0.0%)	1
Absent	138 (100.0%)	164 (100.0%)	
**RAP1B Mutation**			
Present	0 (0.0%)	0 (0.0%)	1
Absent	138 (100.0%)	164 (100.0%)	
**RAPGEF2 Mutation**			
Present	0 (0.0%)	0 (0.0%)	1
Absent	138 (100.0%)	164 (100.0%)	
**RASA1 Mutation**			
Present	6 (4.3%)	10 (6.1%)	0.6457
Absent	132 (95.7%)	154 (93.9%)	
**RASA2 Mutation**			
Present	0 (0.0%)	0 (0.0%)	1
Absent	138 (100.0%)	164 (100.0%)	
**RASGRF1 Mutation**			

Present	0 (0.0%)	0 (0.0%)	1
Absent	138 (100.0%)	164 (100.0%)	
**RASGRF2 Mutation**			
Present	0 (0.0%)	0 (0.0%)	1
Absent	138 (100.0%)	164 (100.0%)	
**RASGRP1 Mutation**			
Present	0 (0.0%)	0 (0.0%)	1
Absent	138 (100.0%)	164 (100.0%)	
**RASGRP2 Mutation**			
Present	0 (0.0%)	0 (0.0%)	1
Absent	138 (100.0%)	164 (100.0%)	
**RASGRP3 Mutation**			
Present	0 (0.0%)	0 (0.0%)	1
Absent	138 (100.0%)	164 (100.0%)	
**RASGRP4 Mutation**			
Present	0 (0.0%)	0 (0.0%)	1
Absent	138 (100.0%)	164 (100.0%)	
**RPS6KA1 Mutation**			
Present	0 (0.0%)	0 (0.0%)	1
Absent	138 (100.0%)	164 (100.0%)	
**RPS6KA2 Mutation**			
Present	0 (0.0%)	0 (0.0%)	1
Absent	138 (100.0%)	164 (100.0%)	
**RPS6KA3 Mutation**			
Present	0 (0.0%)	0 (0.0%)	1

Absent	138 (100.0%)	164 (100.0%)	
**RPS6KA4 Mutation**			
Present	7 (5.1%)	8 (4.9%)	1
Absent	131 (94.9%)	156 (95.1%)	
**RPS6KA5 Mutation**			
Present	0 (0.0%)	0 (0.0%)	1
Absent	138 (100.0%)	164 (100.0%)	
**RPS6KA6 Mutation**			
Present	0 (0.0%)	0 (0.0%)	1
Absent	138 (100.0%)	164 (100.0%)	
**RRAS Mutation**			
Present	1 (0.7%)	2 (1.2%)	1
Absent	137 (99.3%)	162 (98.8%)	
**RRAS2 Mutation**			
Present	1 (0.7%)	3 (1.8%)	0.7405
Absent	137 (99.3%)	161 (98.2%)	
**SOS1 Mutation**			
Present	2 (1.4%)	6 (3.7%)	0.4058
Absent	136 (98.6%)	158 (96.3%)	
**SOS2 Mutation**			
Present	0 (0.0%)	0 (0.0%)	1
Absent	138 (100.0%)	164 (100.0%)	
**SRF Mutation**			
Present	0 (0.0%)	0 (0.0%)	1
Absent	138 (100.0%)	164 (100.0%)	

**STK3 Mutation**			
Present	0 (0.0%)	0 (0.0%)	1
Absent	138 (100.0%)	164 (100.0%)	
**STK4 Mutation**			
Present	0 (0.0%)	0 (0.0%)	1
Absent	138 (100.0%)	164 (100.0%)	
**STMN1 Mutation**			
Present	0 (0.0%)	0 (0.0%)	1
Absent	138 (100.0%)	164 (100.0%)	
**TAOK1 Mutation**			
Present	0 (0.0%)	0 (0.0%)	1
Absent	138 (100.0%)	164 (100.0%)	
**TAOK2 Mutation**			
Present	0 (0.0%)	0 (0.0%)	1
Absent	138 (100.0%)	164 (100.0%)	
**TAOK3 Mutation**			
Present	0 (0.0%)	0 (0.0%)	1
Absent	138 (100.0%)	164 (100.0%)	
**TGFB1 Mutation**			
Present	0 (0.0%)	0 (0.0%)	1
Absent	138 (100.0%)	164 (100.0%)	
**TGFB2 Mutation**			
Present	0 (0.0%)	0 (0.0%)	1
Absent	138 (100.0%)	164 (100.0%)	
**TGFB3 Mutation**			

Present	0 (0.0%)	0 (0.0%)	1
Absent	138 (100.0%)	164 (100.0%)	
**TGFBR1 Mutation**			
Present	4 (2.9%)	6 (3.7%)	0.7591
Absent	134 (97.1%)	158 (96.3%)	
**TGFBR2 Mutation**			
Present	11 (8.0%)	12 (7.3%)	1
Absent	127 (92.0%)	152 (92.7%)	
**TNF Mutation**			
Present	0 (0.0%)	0 (0.0%)	1
Absent	138 (100.0%)	164 (100.0%)	
**TNFRSF1A Mutation**			
Present	0 (0.0%)	0 (0.0%)	1
Absent	138 (100.0%)	164 (100.0%)	
**TP53 Mutation**			
Present	110 (79.7%)	119 (72.6%)	0.1774
Absent	28 (20.3%)	45 (27.4%)	
**TRAF2 Mutation**			
Present	1 (0.7%)	3 (1.8%)	0.6281
Absent	137 (99.3%)	161 (98.2%)	
**TRAF6 Mutation**			
Present	0 (0.0%)	0 (0.0%)	1
Absent	138 (100.0%)	164 (100.0%)	
**ZAK Mutation**			
Present	0 (0.0%)	0 (0.0%)	1

Absent	138 (100.0%)	164 (100.0%)	

Table S2. Alteration rates of JAK/STAT and MAPK pathway-related genes among Early-Onset Colorectal Cancer Hispanic/Latino patients and their Non-Hispanic White counterparts.

Gene	Early Onset H/L n (%)	Early Onset NHW n (%)	p-value
**JAK1 Mutation**			
Present	6 (4.3%)	25 (2.8%)	0.4635
Absent	132 (95.7%)	872 (97.2%)	
**JAK2 Mutation**			
Present	1 (0.7%)	19 (2.1%)	0.5018
Absent	137 (99.3%)	878 (97.9%)	
**JAK3 Mutation**			
Present	4 (2.9%)	23 (2.6%)	0.7742
Absent	134 (97.1%)	874 (97.4%)	
**PIAS1 Mutation**			
Present	0 (0.0%)	1 (0.1%)	1
Absent	138 (100.0%)	896 (99.9%)	
**PIAS2 Mutation**			
Present	0 (0.0%)	1 (0.1%)	1
Absent	138 (100.0%)	896 (99.9%)	
**PIAS3 Mutation**			
Present	0 (0.0%)	0 (0.0%)	1
Absent	138 (100.0%)	897 (100.0%)	
**PTPRC Mutation**			
Present	0 (0.0%)	3 (0.3%)	1

Absent	138 (100.0%)	894 (99.7%)	
**SOCS1 Mutation**			
Present	1 (0.7%)	3 (0.3%)	0.4363
Absent	137 (99.3%)	894 (99.7%)	
**STAT1 Mutation**			
Present	0 (0.0%)	1 (0.1%)	1
Absent	138 (100.0%)	896 (99.9%)	
**STAT3 Mutation**			
Present	2 (1.4%)	14 (1.6%)	1
Absent	136 (98.6%)	883 (98.4%)	
**STAT4 Mutation**			
Present	0 (0.0%)	0 (0.0%)	1
Absent	138 (100.0%)	897 (100.0%)	
**STAT5A Mutation**			
Present	2 (1.4%)	12 (1.3%)	1
Absent	136 (98.6%)	885 (98.7%)	
**STAT5B Mutation**			
Present	6 (4.3%)	16 (1.8%)	0.1037
Absent	132 (95.7%)	881 (98.2%)	
**STAT6 Mutation**			
Present	0 (0.0%)	0 (0.0%)	1
Absent	138 (100.0%)	897 (100.0%)	

Gene	Early Onset H/L n (%)	Early Onset NHW n (%)	p-value
**ACVR1 Mutation**			
Present	4 (2.9%)	11 (1.2%)	0.08248
Absent	134 (97.1%)	886 (98.8%)	
**ACVR1B Mutation**			
Present	0 (0.0%)	1 (0.1%)	1
Absent	138 (100.0%)	896 (99.9%)	
**ACVR1C Mutation**			
Present	0 (0.0%)	1 (0.1%)	1
Absent	138 (100.0%)	896 (99.9%)	
**AKT1 Mutation**			
Present	7 (5.1%)	16 (1.8%)	**0.03319**
Absent	131 (94.9%)	881 (98.2%)	
**AKT2 Mutation**			
Present	1 (0.7%)	7 (0.8%)	1
Absent	137 (99.3%)	890 (99.2%)	
**AKT3 Mutation**			
Present	5 (3.6%)	14 (1.6%)	0.1804
Absent	133 (96.4%)	883 (98.4%)	
**ARRB1 Mutation**			
Present	0 (0.0%)	1 (0.1%)	1
Absent	138 (100.0%)	896 (99.9%)	
**ARRB2 Mutation**			
Present	0 (0.0%)	1 (0.1%)	1
Absent	138 (100.0%)	896 (99.9%)	

**ATF2 Mutation**			
Present	0 (0.0%)	0 (0.0%)	1
Absent	138 (100.0%)	897 (100.0%)	
**ATF4 Mutation**			
Present	0 (0.0%)	0 (0.0%)	1
Absent	138 (100.0%)	897 (100.0%)	
**BDNF Mutation**			
Present	0 (0.0%)	2 (0.2%)	1
Absent	138 (100.0%)	895 (99.8%)	
**BRAF Mutation**			
Present	7 (5.1%)	67 (7.5%)	0.401
Absent	131 (94.9%)	830 (92.5%)	
**CACNA1A Mutation**			
Present	0 (0.0%)	1 (0.1%)	1
Absent	138 (100.0%)	896 (99.9%)	
**CACNA1B Mutation**			
Present	0 (0.0%)	3 (0.3%)	1
Absent	138 (100.0%)	894 (99.7%)	
**CACNA1C Mutation**			
Present	0 (0.0%)	2 (0.2%)	1
Absent	138 (100.0%)	895 (99.8%)	
**CACNA1D Mutation**			
Present	0 (0.0%)	2 (0.2%)	1
Absent	138 (100.0%)	895 (99.8%)	
**CACNA1E Mutation**			

Present	0 (0.0%)	5 (0.6%)	1
Absent	138 (100.0%)	892 (99.4%)	
**CACNAIF Mutation**			
Present	0 (0.0%)	3 (0.3%)	1
Absent	138 (100.0%)	894 (99.7%)	
**CACNA1G Mutation**			
Present	0 (0.0%)	2 (0.2%)	1
Absent	138 (100.0%)	895 (99.8%)	
**CACNA1H Mutation**			
Present	0 (0.0%)	1 (0.1%)	1
Absent	138 (100.0%)	896 (99.9%)	
**CACNA1I Mutation**			
Present	0 (0.0%)	2 (0.2%)	1
Absent	138 (100.0%)	895 (99.8%)	
**CACNA1S Mutation**			
Present	0 (0.0%)	2 (0.2%)	1
Absent	138 (100.0%)	895 (99.8%)	
**CACNA2D1 Mutation**			
Present	0 (0.0%)	1 (0.1%)	1
Absent	138 (100.0%)	896 (99.9%)	
**CACNA2D2 Mutation**			
Present	0 (0.0%)	0 (0.0%)	1
Absent	138 (100.0%)	897 (100.0%)	
**CACNA2D3 Mutation**			
Present	0 (0.0%)	2 (0.2%)	1

Absent	138 (100.0%)	895 (99.8%)	
**CACNA2D4 Mutation**			
Present	0 (0.0%)	2 (0.2%)	1
Absent	138 (100.0%)	895 (99.8%)	
**CACNB1 Mutation**			
Present	0 (0.0%)	2 (0.2%)	1
Absent	138 (100.0%)	895 (99.8%)	
**CACNB2 Mutation**			
Present	0 (0.0%)	0 (0.0%)	1
Absent	138 (100.0%)	897 (100.0%)	
**CACNB3 Mutation**			
Present	0 (0.0%)	1 (0.1%)	1
Absent	138 (100.0%)	896 (99.9%)	
**CACNB4 Mutation**			
Present	0 (0.0%)	1 (0.1%)	1
Absent	138 (100.0%)	896 (99.9%)	
**CACNG1 Mutation**			
Present	0 (0.0%)	0 (0.0%)	1
Absent	138 (100.0%)	897 (100.0%)	
**CACNG2 Mutation**			
Present	0 (0.0%)	1 (0.1%)	1
Absent	138 (100.0%)	896 (99.9%)	
**CACNG3 Mutation**			
Present	0 (0.0%)	1 (0.1%)	1
Absent	138 (100.0%)	896 (99.9%)	

**CACNG4 Mutation**			
Present	0 (0.0%)	0 (0.0%)	1
Absent	138 (100.0%)	897 (100.0%)	
**CACNG5 Mutation**			
Present	0 (0.0%)	1 (0.1%)	1
Absent	138 (100.0%)	896 (99.9%)	
**CACNG6 Mutation**			
Present	0 (0.0%)	1 (0.1%)	1
Absent	138 (100.0%)	896 (99.9%)	
**CACNG7 Mutation**			
Present	0 (0.0%)	0 (0.0%)	1
Absent	138 (100.0%)	897 (100.0%)	
**CACNG8 Mutation**			
Present	0 (0.0%)	0 (0.0%)	1
Absent	138 (100.0%)	897 (100.0%)	
**CASP3 Mutation**			
Present	0 (0.0%)	0 (0.0%)	1
Absent	138 (100.0%)	897 (100.0%)	
**CD14 Mutation**			
Present	0 (0.0%)	0 (0.0%)	1
Absent	138 (100.0%)	897 (100.0%)	
**CDC25B Mutation**			
Present	0 (0.0%)	2 (0.2%)	1
Absent	138 (100.0%)	895 (99.8%)	
**CDC42 Mutation**			

Present	0 (0.0%)	2 (0.2%)	1
Absent	138 (100.0%)	895 (99.8%)	
**CHUK Mutation**			
Present	0 (0.0%)	0 (0.0%)	1
Absent	138 (100.0%)	897 (100.0%)	
**CRK Mutation**			
Present	0 (0.0%)	0 (0.0%)	1
Absent	138 (100.0%)	897 (100.0%)	
**CRKL Mutation**			
Present	0 (0.0%)	7 (0.8%)	0.6031
Absent	138 (100.0%)	890 (99.2%)	
**DAXX Mutation**			
Present	4 (2.9%)	14 (1.6%)	0.2845
Absent	134 (97.1%)	883 (98.4%)	
**DDIT3 Mutation**			
Present	0 (0.0%)	0 (0.0%)	1
Absent	138 (100.0%)	897 (100.0%)	
**DUSP1 Mutation**			
Present	0 (0.0%)	0 (0.0%)	1
Absent	138 (100.0%)	897 (100.0%)	
**DUSP10 Mutation**			
Present	0 (0.0%)	0 (0.0%)	1
Absent	138 (100.0%)	897 (100.0%)	
**DUSP14 Mutation**			
Present	0 (0.0%)	0 (0.0%)	1

Absent	138 (100.0%)	897 (100.0%)	
**DUSP16 Mutation**			
Present	0 (0.0%)	0 (0.0%)	1
Absent	138 (100.0%)	897 (100.0%)	
**DUSP2 Mutation**			
Present	0 (0.0%)	0 (0.0%)	1
Absent	138 (100.0%)	897 (100.0%)	
**DUSP3 Mutation**			
Present	0 (0.0%)	1 (0.1%)	1
Absent	138 (100.0%)	896 (99.9%)	
**DUSP4 Mutation**			
Present	0 (0.0%)	3 (0.3%)	1
Absent	138 (100.0%)	894 (99.7%)	
**DUSP5 Mutation**			
Present	0 (0.0%)	0 (0.0%)	1
Absent	138 (100.0%)	897 (100.0%)	
**DUSP6 Mutation**			
Present	0 (0.0%)	0 (0.0%)	1
Absent	138 (100.0%)	897 (100.0%)	
**DUSP7 Mutation**			
Present	0 (0.0%)	1 (0.1%)	1
Absent	138 (100.0%)	896 (99.9%)	
**DUSP8 Mutation**			
Present	0 (0.0%)	0 (0.0%)	1
Absent	138 (100.0%)	897 (100.0%)	

**DUSP9 Mutation**			
Present	0 (0.0%)	0 (0.0%)	1
Absent	138 (100.0%)	897 (100.0%)	
**ECSIT Mutation**			
Present	0 (0.0%)	0 (0.0%)	1
Absent	138 (100.0%)	897 (100.0%)	
**EGF Mutation**			
Present	0 (0.0%)	1 (0.1%)	1
Absent	138 (100.0%)	896 (99.9%)	
**EGFR Mutation**			
Present	5 (3.6%)	16 (1.8%)	0.2702
Absent	133 (96.4%)	881 (98.2%)	
**ELK1 Mutation**			
Present	0 (0.0%)	0 (0.0%)	1
Absent	138 (100.0%)	897 (100.0%)	
**ELK4 Mutation**			
Present	0 (0.0%)	2 (0.2%)	1
Absent	138 (100.0%)	895 (99.8%)	
**FAS Mutation**			
Present	1 (0.7%)	2 (0.2%)	0.3493
Absent	137 (99.3%)	895 (99.8%)	
**FASLG Mutation**			
Present	0 (0.0%)	0 (0.0%)	1
Absent	138 (100.0%)	897 (100.0%)	
**FGF1 Mutation**			

Present	0 (0.0%)	0 (0.0%)	1
Absent	138 (100.0%)	897 (100.0%)	
**FGF10 Mutation**			
Present	0 (0.0%)	1 (0.1%)	1
Absent	138 (100.0%)	896 (99.9%)	
**FGF11 Mutation**			
Present	0 (0.0%)	1 (0.1%)	1
Absent	138 (100.0%)	896 (99.9%)	
**FGF12 Mutation**			
Present	0 (0.0%)	0 (0.0%)	1
Absent	138 (100.0%)	897 (100.0%)	
**FGF13 Mutation**			
Present	0 (0.0%)	1 (0.1%)	1
Absent	138 (100.0%)	896 (99.9%)	
**FGF14 Mutation**			
Present	0 (0.0%)	0 (0.0%)	1
Absent	138 (100.0%)	897 (100.0%)	
**FGF16 Mutation**			
Present	0 (0.0%)	0 (0.0%)	1
Absent	138 (100.0%)	897 (100.0%)	
**FGF17 Mutation**			
Present	0 (0.0%)	1 (0.1%)	1
Absent	138 (100.0%)	896 (99.9%)	
**FGF18 Mutation**			
Present	0 (0.0%)	0 (0.0%)	1

Absent	138 (100.0%)	897 (100.0%)	
**FGF19 Mutation**			
Present	1 (0.7%)	8 (0.9%)	1
Absent	137 (99.3%)	889 (99.1%)	
**FGF2 Mutation**			
Present	0 (0.0%)	0 (0.0%)	1
Absent	138 (100.0%)	897 (100.0%)	
**FGF20 Mutation**			
Present	0 (0.0%)	0 (0.0%)	1
Absent	138 (100.0%)	897 (100.0%)	
**FGF21 Mutation**			
Present	0 (0.0%)	0 (0.0%)	1
Absent	138 (100.0%)	897 (100.0%)	
**FGF22 Mutation**			
Present	0 (0.0%)	0 (0.0%)	1
Absent	138 (100.0%)	897 (100.0%)	
**FGF23 Mutation**			
Present	0 (0.0%)	2 (0.2%)	1
Absent	138 (100.0%)	895 (99.8%)	
**FGF3 Mutation**			
Present	2 (1.4%)	12 (1.3%)	1
Absent	136 (98.6%)	885 (98.7%)	
**FGF4 Mutation**			
Present	0 (0.0%)	5 (0.6%)	1
Absent	138 (100.0%)	892 (99.4%)	

**FGF5 Mutation**			
Present	0 (0.0%)	0 (0.0%)	1
Absent	138 (100.0%)	897 (100.0%)	
**FGF6 Mutation**			
Present	0 (0.0%)	0 (0.0%)	1
Absent	138 (100.0%)	897 (100.0%)	
**FGF7 Mutation**			
Present	0 (0.0%)	0 (0.0%)	1
Absent	138 (100.0%)	897 (100.0%)	
**FGF8 Mutation**			
Present	0 (0.0%)	0 (0.0%)	1
Absent	138 (100.0%)	897 (100.0%)	
**FGF9 Mutation**			
Present	0 (0.0%)	0 (0.0%)	1
Absent	138 (100.0%)	897 (100.0%)	
**FGFR1 Mutation**			
Present	2 (1.4%)	22 (2.5%)	0.7598
Absent	136 (98.6%)	875 (97.5%)	
**FGFR2 Mutation**			
Present	5 (3.6%)	21 (2.3%)	0.546
Absent	133 (96.4%)	876 (97.7%)	
**FGFR3 Mutation**			
Present	5 (3.6%)	24 (2.7%)	0.7256
Absent	133 (96.4%)	873 (97.3%)	
**FGFR4 Mutation**			

Present	3 (2.2%)	17 (1.9%)	0.7415
Absent	135 (97.8%)	880 (98.1%)	
**FLNA Mutation**			
Present	0 (0.0%)	0 (0.0%)	1
Absent	138 (100.0%)	897 (100.0%)	
**FLNB Mutation**			
Present	0 (0.0%)	1 (0.1%)	1
Absent	138 (100.0%)	896 (99.9%)	
**FLNC Mutation**			
Present	0 (0.0%)	4 (0.4%)	1
Absent	138 (100.0%)	893 (99.6%)	
**FOS Mutation**			
Present	0 (0.0%)	1 (0.1%)	1
Absent	138 (100.0%)	896 (99.9%)	
**GADD45A Mutation**			
Present	0 (0.0%)	0 (0.0%)	1
Absent	138 (100.0%)	897 (100.0%)	
**GADD45B Mutation**			
Present	0 (0.0%)	0 (0.0%)	1
Absent	138 (100.0%)	897 (100.0%)	
**GADD45G Mutation**			
Present	0 (0.0%)	0 (0.0%)	1
Absent	138 (100.0%)	897 (100.0%)	
**GNA12 Mutation**			
Present	0 (0.0%)	0 (0.0%)	1

Absent	138 (100.0%)	897 (100.0%)	
**GNG12 Mutation**			
Present	0 (0.0%)	1 (0.1%)	1
Absent	138 (100.0%)	896 (99.9%)	
**GRB2 Mutation**			
Present	0 (0.0%)	0 (0.0%)	1
Absent	138 (100.0%)	897 (100.0%)	
**HRAS Mutation**			
Present	2 (1.4%)	8 (0.9%)	0.631
Absent	136 (98.6%)	889 (99.1%)	
**IKBKB Mutation**			
Present	0 (0.0%)	0 (0.0%)	1
Absent	138 (100.0%)	897 (100.0%)	
**IKBKG Mutation**			
Present	0 (0.0%)	0 (0.0%)	1
Absent	138 (100.0%)	897 (100.0%)	
**IL1A Mutation**			
Present	0 (0.0%)	0 (0.0%)	1
Absent	138 (100.0%)	897 (100.0%)	
**IL1B Mutation**			
Present	0 (0.0%)	0 (0.0%)	1
Absent	138 (100.0%)	897 (100.0%)	
**IL1R1 Mutation**			
Present	0 (0.0%)	2 (0.2%)	1
Absent	138 (100.0%)	895 (99.8%)	

**IL1R2 Mutation**			
Present	0 (0.0%)	0 (0.0%)	1
Absent	138 (100.0%)	897 (100.0%)	
**JUN Mutation**			
Present	3 (2.2%)	14 (1.6%)	
Absent	135 (97.8%)	883 (98.4%)	
**JUND Mutation**			
Present	0 (0.0%)	0 (0.0%)	1
Absent	138 (100.0%)	897 (100.0%)	
**KRAS Mutation**			
Present	51 (37.0%)	371 (41.4%)	0.4102
Absent	86 (62.3%)	526 (58.6%)	
**MAP2K1 Mutation**			
Present	5 (3.6%)	16 (1.8%)	0.2702
Absent	133 (96.4%)	881 (98.2%)	
**MAP2K2 Mutation**			
Present	3 (2.2%)	6 (0.7%)	0.1063
Absent	135 (97.8%)	891 (99.3%)	
**MAP2K3 Mutation**			
Present	0 (0.0%)	0 (0.0%)	1
Absent	138 (100.0%)	897 (100.0%)	
**MAP2K4 Mutation**			
Present	6 (4.3%)	21 (2.3%)	0.2757
Absent	132 (95.7%)	876 (97.7%)	
**MAP2K5 Mutation**			

Present	0 (0.0%)	0 (0.0%)	1
Absent	138 (100.0%)	897 (100.0%)	
**MAP2K6 Mutation**			
Present	0 (0.0%)	0 (0.0%)	1
Absent	138 (100.0%)	897 (100.0%)	
**MAP2K7 Mutation**			
Present	0 (0.0%)	3 (0.3%)	1
Absent	138 (100.0%)	894 (99.7%)	
**MAP3K1 Mutation**			
Present	7 (5.1%)	28 (3.1%)	0.3537
Absent	131 (94.9%)	869 (96.9%)	
**MAP3K10 Mutation**			
Present	0 (0.0%)	0 (0.0%)	1
Absent	138 (100.0%)	897 (100.0%)	
**MAP3K12 Mutation**			
Present	0 (0.0%)	0 (0.0%)	1
Absent	138 (100.0%)	897 (100.0%)	
**MAP3K13 Mutation**			
Present	2 (1.4%)	20 (2.2%)	0.7566
Absent	136 (98.6%)	877 (97.8%)	
**MAP3K14 Mutation**			
Present	0 (0.0%)	1 (0.1%)	1
Absent	138 (100.0%)	896 (99.9%)	
**MAP3K2 Mutation**			
Present	0 (0.0%)	0 (0.0%)	1

Absent	138 (100.0%)	897 (100.0%)	
**MAP3K3 Mutation**			
Present	0 (0.0%)	1 (0.1%)	1
Absent	138 (100.0%)	896 (99.9%)	
**MAP3K4 Mutation**			
Present	0 (0.0%)	1 (0.1%)	1
Absent	138 (100.0%)	896 (99.9%)	
**MAP3K5 Mutation**			
Present	0 (0.0%)	1 (0.1%)	1
Absent	138 (100.0%)	896 (99.9%)	
**MAP3K6 Mutation**			
Present	1 (0.7%)	1 (0.1%)	0.249
Absent	137 (99.3%)	896 (99.9%)	
**MAP3K7 Mutation**			
Present	0 (0.0%)	0 (0.0%)	1
Absent	138 (100.0%)	897 (100.0%)	
**MAP3K8 Mutation**			
Present	0 (0.0%)	0 (0.0%)	1
Absent	138 (100.0%)	897 (100.0%)	
**MAP4K1 Mutation**			
Present	0 (0.0%)	1 (0.1%)	1
Absent	138 (100.0%)	896 (99.9%)	
**MAP4K2 Mutation**			
Present	0 (0.0%)	0 (0.0%)	1
Absent	138 (100.0%)	897 (100.0%)	

**MAP4K3 Mutation**			
Present	0 (0.0%)	1 (0.1%)	1
Absent	138 (100.0%)	896 (99.9%)	
**MAP4K4 Mutation**			
Present	1 (0.7%)	2 (0.2%)	0.3493
Absent	137 (99.3%)	895 (99.8%)	
**MAPK1 Mutation**			
Present	2 (1.4%)	7 (0.8%)	0.3423
Absent	136 (98.6%)	890 (99.2%)	
**MAPK10 Mutation**			
Present	0 (0.0%)	1 (0.1%)	1
Absent	138 (100.0%)	896 (99.9%)	
**MAPK11 Mutation**			
Present	0 (0.0%)	0 (0.0%)	1
Absent	138 (100.0%)	897 (100.0%)	
**MAPK12 Mutation**			
Present	0 (0.0%)	0 (0.0%)	1
Absent	138 (100.0%)	897 (100.0%)	
**MAPK13 Mutation**			
Present	0 (0.0%)	1 (0.1%)	1
Absent	138 (100.0%)	896 (99.9%)	
**MAPK14 Mutation**			
Present	0 (0.0%)	1 (0.1%)	1
Absent	138 (100.0%)	896 (99.9%)	
**MAPK3 Mutation**			

Present	5 (3.6%)	6 (0.7%)	**0.006833**
Absent	133 (96.4%)	891 (99.3%)	
**MAPK7 Mutation**			
Present	0 (0.0%)	0 (0.0%)	1
Absent	138 (100.0%)	897 (100.0%)	
**MAPK8 Mutation**			
Present	0 (0.0%)	1 (0.1%)	1
Absent	138 (100.0%)	896 (99.9%)	
**MAPK8IP1 Mutation**			
Present	0 (0.0%)	1 (0.1%)	1
Absent	138 (100.0%)	896 (99.9%)	
**MAPK8IP2 Mutation**			
Present	0 (0.0%)	1 (0.1%)	1
Absent	138 (100.0%)	896 (99.9%)	
**MAPK8IP3 Mutation**			
Present	0 (0.0%)	1 (0.1%)	1
Absent	138 (100.0%)	896 (99.9%)	
**MAPK9 Mutation**			
Present	0 (0.0%)	0 (0.0%)	1
Absent	138 (100.0%)	897 (100.0%)	
**MAPKAPK2 Mutation**			
Present	0 (0.0%)	0 (0.0%)	1
Absent	138 (100.0%)	897 (100.0%)	
**MAPKAPK3 Mutation**			
Present	0 (0.0%)	0 (0.0%)	1

Absent	138 (100.0%)	897 (100.0%)	
**MAPKAPK5 Mutation**			
Present	0 (0.0%)	0 (0.0%)	1
Absent	138 (100.0%)	897 (100.0%)	
**MAPT Mutation**			
Present	0 (0.0%)	1 (0.1%)	1
Absent	138 (100.0%)	896 (99.9%)	
**MAX Mutation**			
Present	0 (0.0%)	6 (0.7%)	1
Absent	138 (100.0%)	891 (99.3%)	
**MEF2C Mutation**			
Present	0 (0.0%)	0 (0.0%)	1
Absent	138 (100.0%)	897 (100.0%)	
**MKNK1 Mutation**			
Present	0 (0.0%)	0 (0.0%)	1
Absent	138 (100.0%)	897 (100.0%)	
**MKNK2 Mutation**			
Present	0 (0.0%)	0 (0.0%)	1
Absent	138 (100.0%)	897 (100.0%)	
**MOS Mutation**			
Present	0 (0.0%)	1 (0.1%)	1
Absent	138 (100.0%)	896 (99.9%)	
**MRAS Mutation**			
Present	0 (0.0%)	0 (0.0%)	1
Absent	138 (100.0%)	897 (100.0%)	

**MYC Mutation**			
Present	1 (0.7%)	6 (0.7%)	1
Absent	137 (99.3%)	891 (99.3%)	
**NF1 Mutation**			
Present	16 (11.6%)	55 (6.1%)	**0.02907**
Absent	122 (88.4%)	842 (93.9%)	
**NFATC2 Mutation**			
Present	0 (0.0%)	1 (0.1%)	1
Absent	138 (100.0%)	896 (99.9%)	
**NFATC4 Mutation**			
Present	0 (0.0%)	2 (0.2%)	1
Absent	138 (100.0%)	895 (99.8%)	
**NFKB1 Mutation**			
Present	0 (0.0%)	1 (0.1%)	1
Absent	138 (100.0%)	896 (99.9%)	
**NFKB2 Mutation**			
Present	0 (0.0%)	0 (0.0%)	1
Absent	138 (100.0%)	897 (100.0%)	
**NLK Mutation**			
Present	0 (0.0%)	0 (0.0%)	1
Absent	138 (100.0%)	897 (100.0%)	
**NR4A1 Mutation**			
Present	0 (0.0%)	0 (0.0%)	1
Absent	138 (100.0%)	897 (100.0%)	
**NRAS Mutation**			

Present	8 (5.8%)	25 (2.8%)	0.1067
Absent	130 (94.2%)	872 (97.2%)	
**NTF3 Mutation**			
Present	0 (0.0%)	0 (0.0%)	1
Absent	138 (100.0%)	897 (100.0%)	
**NTRK1 Mutation**			
Present	5 (3.6%)	22 (2.5%)	0.6056
Absent	133 (96.4%)	875 (97.5%)	
**NTRK2 Mutation**			
Present	4 (2.9%)	19 (2.1%)	0.534
Absent	134 (97.1%)	878 (97.9%)	
**PAK1 Mutation**			
Present	3 (2.2%)	12 (1.3%)	0.4376
Absent	135 (97.8%)	885 (98.7%)	
**PAK2 Mutation**			
Present	0 (0.0%)	1 (0.1%)	1
Absent	138 (100.0%)	896 (99.9%)	
**PDGFA Mutation**			
Present	0 (0.0%)	0 (0.0%)	1
Absent	138 (100.0%)	897 (100.0%)	
**PDGFB Mutation**			
Present	0 (0.0%)	1 (0.1%)	1
Absent	138 (100.0%)	896 (99.9%)	
**PDGFRA Mutation**			
Present	8 (5.8%)	36 (4.0%)	0.4591

Absent	130 (94.2%)	861 (96.0%)	
**PDGFRB Mutation**			
Present	8 (5.8%)	19 (2.1%)	**0.02527**
Absent	130 (94.2%)	878 (97.9%)	
**PLA2G10 Mutation**			
Present	0 (0.0%)	0 (0.0%)	1
Absent	138 (100.0%)	897 (100.0%)	
**PLA2G12A Mutation**			
Present	0 (0.0%)	0 (0.0%)	1
Absent	138 (100.0%)	897 (100.0%)	
**PLA2G12B Mutation**			
Present	0 (0.0%)	0 (0.0%)	1
Absent	138 (100.0%)	897 (100.0%)	
**PLA2G1B Mutation**			
Present	0 (0.0%)	0 (0.0%)	1
Absent	138 (100.0%)	897 (100.0%)	
**PLA2G2A Mutation**			
Present	0 (0.0%)	0 (0.0%)	1
Absent	138 (100.0%)	897 (100.0%)	
**PLA2G2D Mutation**			
Present	0 (0.0%)	0 (0.0%)	1
Absent	138 (100.0%)	897 (100.0%)	
**PLA2G2E Mutation**			
Present	0 (0.0%)	0 (0.0%)	1
Absent	138 (100.0%)	897 (100.0%)	

**PLA2G2F Mutation**			
Present	0 (0.0%)	0 (0.0%)	1
Absent	138 (100.0%)	897 (100.0%)	
**PLA2G3 Mutation**			
Present	0 (0.0%)	0 (0.0%)	1
Absent	138 (100.0%)	897 (100.0%)	
**PLA2G4A Mutation**			
Present	0 (0.0%)	0 (0.0%)	1
Absent	138 (100.0%)	897 (100.0%)	
**PLA2G5 Mutation**			
Present	0 (0.0%)	0 (0.0%)	1
Absent	138 (100.0%)	897 (100.0%)	
**PLA2G6 Mutation**			
Present	0 (0.0%)	0 (0.0%)	1
Absent	138 (100.0%)	897 (100.0%)	
**PPM1A Mutation**			
Present	0 (0.0%)	0 (0.0%)	1
Absent	138 (100.0%)	897 (100.0%)	
**PPM1B Mutation**			
Present	0 (0.0%)	1 (0.1%)	1
Absent	138 (100.0%)	896 (99.9%)	
**PPP3CA Mutation**			
Present	0 (0.0%)	0 (0.0%)	1
Absent	138 (100.0%)	897 (100.0%)	
**PPP3CB Mutation**			

Present	0 (0.0%)	1 (0.1%)	1
Absent	138 (100.0%)	896 (99.9%)	
**PPP3CC Mutation**			
Present	0 (0.0%)	0 (0.0%)	1
Absent	138 (100.0%)	897 (100.0%)	
**PPP3R1 Mutation**			
Present	0 (0.0%)	0 (0.0%)	1
Absent	138 (100.0%)	897 (100.0%)	
**PPP3R2 Mutation**			
Present	0 (0.0%)	1 (0.1%)	1
Absent	138 (100.0%)	896 (99.9%)	
**PPP5C Mutation**			
Present	0 (0.0%)	1 (0.1%)	1
Absent	138 (100.0%)	896 (99.9%)	
**PRKACA Mutation**			
Present	0 (0.0%)	0 (0.0%)	1
Absent	138 (100.0%)	897 (100.0%)	
**PRKACB Mutation**			
Present	0 (0.0%)	0 (0.0%)	1
Absent	138 (100.0%)	897 (100.0%)	
**PRKACG Mutation**			
Present	0 (0.0%)	2 (0.2%)	1
Absent	138 (100.0%)	895 (99.8%)	
**PRKCA Mutation**			
Present	0 (0.0%)	0 (0.0%)	1

Absent	138 (100.0%)	897 (100.0%)	
**PRKCG Mutation**			
Present	0 (0.0%)	1 (0.1%)	1
Absent	138 (100.0%)	896 (99.9%)	
**PRKX Mutation**			
Present	0 (0.0%)	0 (0.0%)	1
Absent	138 (100.0%)	897 (100.0%)	
**PRKY Mutation**			
Present	0 (0.0%)	0 (0.0%)	1
Absent	138 (100.0%)	897 (100.0%)	
**PTPN5 Mutation**			
Present	0 (0.0%)	1 (0.1%)	1
Absent	138 (100.0%)	896 (99.9%)	
**PTPN7 Mutation**			
Present	0 (0.0%)	1 (0.1%)	1
Absent	138 (100.0%)	896 (99.9%)	
**PTPRR Mutation**			
Present	0 (0.0%)	2 (0.2%)	1
Absent	138 (100.0%)	895 (99.8%)	
**RAC1 Mutation**			
Present	1 (0.7%)	8 (0.9%)	1
Absent	137 (99.3%)	889 (99.1%)	
**RAC2 Mutation**			
Present	1 (0.7%)	7 (0.8%)	1
Absent	137 (99.3%)	890 (99.2%)	

**RAC3 Mutation**			
Present	0 (0.0%)	3 (0.3%)	1
Absent	138 (100.0%)	894 (99.7%)	
**RAF1 Mutation**			
Present	3 (2.2%)	24 (2.7%)	1
Absent	135 (97.8%)	873 (97.3%)	
**RAP1A Mutation**			
Present	0 (0.0%)	1 (0.1%)	1
Absent	138 (100.0%)	896 (99.9%)	
**RAP1B Mutation**			
Present	0 (0.0%)	0 (0.0%)	1
Absent	138 (100.0%)	897 (100.0%)	
**RAPGEF2 Mutation**			
Present	0 (0.0%)	2 (0.2%)	1
Absent	138 (100.0%)	895 (99.8%)	
**RASA1 Mutation**			
Present	6 (4.3%)	34 (3.8%)	0.937
Absent	132 (95.7%)	863 (96.2%)	
**RASA2 Mutation**			
Present	0 (0.0%)	1 (0.1%)	1
Absent	138 (100.0%)	896 (99.9%)	
**RASGRF1 Mutation**			
Present	0 (0.0%)	3 (0.3%)	1
Absent	138 (100.0%)	894 (99.7%)	
**RASGRF2 Mutation**			

Present	0 (0.0%)	1 (0.1%)	1
Absent	138 (100.0%)	896 (99.9%)	
**RASGRP1 Mutation**			
Present	0 (0.0%)	1 (0.1%)	1
Absent	138 (100.0%)	896 (99.9%)	
**RASGRP2 Mutation**			
Present	0 (0.0%)	1 (0.1%)	1
Absent	138 (100.0%)	896 (99.9%)	
**RASGRP3 Mutation**			
Present	0 (0.0%)	1 (0.1%)	1
Absent	138 (100.0%)	896 (99.9%)	
**RASGRP4 Mutation**			
Present	0 (0.0%)	0 (0.0%)	1
Absent	138 (100.0%)	897 (100.0%)	
**RPS6KA1 Mutation**			
Present	0 (0.0%)	1 (0.1%)	1
Absent	138 (100.0%)	896 (99.9%)	
**RPS6KA2 Mutation**			
Present	0 (0.0%)	1 (0.1%)	1
Absent	138 (100.0%)	896 (99.9%)	
**RPS6KA3 Mutation**			
Present	0 (0.0%)	1 (0.1%)	1
Absent	138 (100.0%)	896 (99.9%)	
**RPS6KA4 Mutation**			
Present	7 (5.1%)	18 (2.0%)	0.05929

Absent	131 (94.9%)	879 (98.0%)	
**RPS6KA5 Mutation**			
Present	0 (0.0%)	0 (0.0%)	1
Absent	138 (100.0%)	897 (100.0%)	
**RPS6KA6 Mutation**			
Present	0 (0.0%)	1 (0.1%)	1
Absent	138 (100.0%)	896 (99.9%)	
**RRAS Mutation**			
Present	1 (0.7%)	6 (0.7%)	1
Absent	137 (99.3%)	891 (99.3%)	
**RRAS2 Mutation**			
Present	1 (0.7%)	5 (0.6%)	0.5772
Absent	137 (99.3%)	892 (99.4%)	
**SOS1 Mutation**			
Present	2 (1.4%)	28 (3.1%)	0.4136
Absent	136 (98.6%)	869 (96.9%)	
**SOS2 Mutation**			
Present	0 (0.0%)	0 (0.0%)	1
Absent	138 (100.0%)	897 (100.0%)	
**SRF Mutation**			
Present	0 (0.0%)	0 (0.0%)	1
Absent	138 (100.0%)	897 (100.0%)	
**STK3 Mutation**			
Present	0 (0.0%)	0 (0.0%)	1
Absent	138 (100.0%)	897 (100.0%)	

**STK4 Mutation**			
Present	0 (0.0%)	0 (0.0%)	1
Absent	138 (100.0%)	897 (100.0%)	
**STMN1 Mutation**			
Present	0 (0.0%)	0 (0.0%)	1
Absent	138 (100.0%)	897 (100.0%)	
**TAOK1 Mutation**			
Present	0 (0.0%)	1 (0.1%)	1
Absent	138 (100.0%)	896 (99.9%)	
**TAOK2 Mutation**			
Present	0 (0.0%)	1 (0.1%)	1
Absent	138 (100.0%)	896 (99.9%)	
**TAOK3 Mutation**			
Present	0 (0.0%)	2 (0.2%)	1
Absent	138 (100.0%)	895 (99.8%)	
**TGFB1 Mutation**			
Present	0 (0.0%)	0 (0.0%)	1
Absent	138 (100.0%)	897 (100.0%)	
**TGFB2 Mutation**			
Present	0 (0.0%)	2 (0.2%)	1
Absent	138 (100.0%)	895 (99.8%)	
**TGFB3 Mutation**			
Present	0 (0.0%)	1 (0.1%)	1
Absent	138 (100.0%)	896 (99.9%)	
**TGFBR1 Mutation**			

Present	4 (2.9%)	21 (2.3%)	0.7634
Absent	134 (97.1%)	876 (97.7%)	
**TGFBR2 Mutation**			
Present	11 (8.0%)	38 (4.2%)	0.08765
Absent	127 (92.0%)	859 (95.8%)	
**TNF Mutation**			
Present	0 (0.0%)	0 (0.0%)	1
Absent	138 (100.0%)	897 (100.0%)	
**TNFRSF1A Mutation**			
Present	0 (0.0%)	0 (0.0%)	1
Absent	138 (100.0%)	897 (100.0%)	
**TP53 Mutation**			
Present	110 (79.7%)	683 (76.1%)	0.4158
Absent	28 (20.3%)	214 (23.9%)	
**TRAF2 Mutation**			
Present	1 (0.7%)	15 (1.7%)	0.7102
Absent	137 (99.3%)	882 (98.3%)	
**TRAF6 Mutation**			
Present	0 (0.0%)	0 (0.0%)	1
Absent	138 (100.0%)	897 (100.0%)	
**ZAK Mutation**			
Present	0 (0.0%)	0 (0.0%)	1
Absent	138 (100.0%)	897 (100.0%)	

Table S3. Nature of the mutations of MAPK pathway alterations in Early-Onset Colorectal Cancer (EOCRC) among Hispanic/Latino (H/L).

	Early-Onset Hispanic/Latino Samples						
	**Frame Shift Deletion**	**Frame Shift Insertion**	**In Frame Deletion**	**Missense Mutation**	**Nonsense Mutation**	**Splice Site**	**Translation Start Site**
**ACVR1**	0.0%	0.0%	0.0%	100.0%	0.0%	0.0%	0.0%
**AKT1**	0.0%	0.0%	16.7%	83.3%	0.0%	0.0%	0.0%
**AKT2**	100.0%	0.0%	0.0%	0.0%	0.0%	0.0%	0.0%
**AKT3**	0.0%	0.0%	0.0%	60.0%	40.0%	0.0%	0.0%
**BRAF**	0.0%	0.0%	0.0%	100.0%	0.0%	0.0%	0.0%
**CRKL**	0.0%	0.0%	0.0%	100.0%	0.0%	0.0%	0.0%
**DAXX**	28.6%	0.0%	0.0%	71.4%	0.0%	0.0%	0.0%
**EGFR**	20.0%	0.0%	0.0%	60.0%	0.0%	20.0%	0.0%
**FGF19**	0.0%	0.0%	0.0%	100.0%	0.0%	0.0%	0.0%
**FGF3**	50.0%	0.0%	0.0%	50.0%	0.0%	0.0%	0.0%
**FGFR1**	0.0%	0.0%	0.0%	100.0%	0.0%	0.0%	0.0%
**FGFR2**	0.0%	0.0%	0.0%	77.8%	22.2%	0.0%	0.0%
**FGFR3**	0.0%	0.0%	0.0%	100.0%	0.0%	0.0%	0.0%
**FGFR4**	33.3%	0.0%	0.0%	50.0%	0.0%	16.7%	0.0%
**HRAS**	0.0%	0.0%	0.0%	100.0%	0.0%	0.0%	0.0%
**JUN**	40.0%	40.0%	0.0%	20.0%	0.0%	0.0%	0.0%
**KRAS**	0.0%	0.0%	0.0%	100.0%	0.0%	0.0%	0.0%
**MAP2K1**	0.0%	0.0%	0.0%	100.0%	0.0%	0.0%	0.0%
**MAP2K2**	0.0%	0.0%	0.0%	100.0%	0.0%	0.0%	0.0%
**MAP2K4**	0.0%	0.0%	0.0%	50.0%	33.3%	16.7%	0.0%
**MAP3K1**	0.0%	10.0%	0.0%	90.0%	0.0%	0.0%	0.0%

**MAP3K13**	0.0%	0.0%	0.0%	100.0%	0.0%	0.0%	0.0%
**MAPK1**	0.0%	0.0%	0.0%	100.0%	0.0%	0.0%	0.0%
**MAPK3**	0.0%	0.0%	0.0%	100.0%	0.0%	0.0%	0.0%
**NF1**	21.2%	12.1%	0.0%	48.5%	9.1%	9.1%	0.0%
**NRAS**	0.0%	0.0%	0.0%	100.0%	0.0%	0.0%	0.0%
**NTRK1**	0.0%	0.0%	0.0%	100.0%	0.0%	0.0%	0.0%
**NTRK2**	0.0%	0.0%	0.0%	100.0%	0.0%	0.0%	0.0%
**PAK1**	0.0%	0.0%	0.0%	80.0%	20.0%	0.0%	0.0%
**PDGFRA**	0.0%	0.0%	0.0%	92.9%	0.0%	7.1%	0.0%
**PDGFRB**	0.0%	0.0%	0.0%	90.0%	10.0%	0.0%	0.0%
**RAC1**	0.0%	0.0%	0.0%	100.0%	0.0%	0.0%	0.0%
**RAC2**	0.0%	0.0%	0.0%	100.0%	0.0%	0.0%	0.0%
**RAF1**	0.0%	0.0%	0.0%	100.0%	0.0%	0.0%	0.0%
**RASA1**	50.0%	0.0%	0.0%	37.5%	12.5%	0.0%	0.0%
**RPS6KA4**	14.3%	0.0%	0.0%	85.7%	0.0%	0.0%	0.0%
**RRAS**	0.0%	0.0%	0.0%	100.0%	0.0%	0.0%	0.0%
**RRAS2**	0.0%	0.0%	0.0%	100.0%	0.0%	0.0%	0.0%
**SOS1**	0.0%	0.0%	0.0%	50.0%	50.0%	0.0%	0.0%
**TGFBR1**	0.0%	0.0%	0.0%	85.7%	0.0%	0.0%	14.3%
**TGFBR2**	41.7%	0.0%	0.0%	58.3%	0.0%	0.0%	0.0%
**TP53**	6.4%	4.0%	0.0%	64.8%	18.4%	6.4%	0.0%
**TRAF2**	100.0%	0.0%	0.0%	0.0%	0.0%	0.0%	0.0%

Table S4. Nature of the mutations of JAK/STAT pathway alterations in Early-Onset Colorectal Cancer (EOCRC) among Hispanic/Latino (H/L).

	Early-Onset Hispanic/Latino Samples				
	**Frame Shift Deletion**	**Frame Shift Insertion**	**Missense Mutation**	**Nonsense Mutation**	**Splice Site**
**JAK1**	12.5%	6.3%	31.3%	12.5%	37.5%
**JAK2**	0.0%	0.0%	100.0%	0.0%	0.0%
**JAK3**	0.0%	0.0%	75.0%	25.0%	0.0%
**SOCS1**	0.0%	0.0%	100.0%	0.0%	0.0%
**STAT3**	0.0%	50.0%	50.0%	0.0%	0.0%
**STAT5A**	50.0%	50.0%	0.0%	0.0%	0.0%
**STAT5B**	50.0%	33.3%	16.7%	0.0%	0.0%

Table S5. Nature of the mutations of MAPK pathway alterations in Late-Onset Colorectal Cancer (LOCRC) among Hispanic/Latino (H/L).

	Late-Onset Hispanic/Latino Samples								
	**Frame Shift Deletion**	**Frame Shift Insertion**	**In Frame Deletion**	**In Frame Insertion**	**Missense Mutation**	**Nonsense Mutation**	**Splice Region**	**Splic e Site**	**Translation Start Site**
AKT1	0.0%	0.0%	0.0%	0.0%	100.0%	0.0%	0.0%	0.0%	0.0%
AKT2	50.0%	0.0%	0.0%	0.0%	50.0%	0.0%	0.0%	0.0%	0.0%
AKT3	40.0%	0.0%	0.0%	0.0%	40.0%	20.0%	0.0%	0.0%	0.0%
BRAF	0.0%	0.0%	0.0%	0.0%	100.0%	0.0%	0.0%	0.0%	0.0%
CACNA1A	66.7%	0.0%	0.0%	0.0%	33.3%	0.0%	0.0%	0.0%	0.0%
CACNA1E	0.0%	0.0%	0.0%	0.0%	100.0%	0.0%	0.0%	0.0%	0.0%
CACNA1H	50.0%	0.0%	0.0%	0.0%	50.0%	0.0%	0.0%	0.0%	0.0%
CACNA1I	100.0%	0.0%	0.0%	0.0%	0.0%	0.0%	0.0%	0.0%	0.0%
CACNA2D 1	0.0%	0.0%	0.0%	0.0%	100.0%	0.0%	0.0%	0.0%	0.0%
CACNA2D 3	0.0%	0.0%	0.0%	0.0%	100.0%	0.0%	0.0%	0.0%	0.0%
CACNG3	0.0%	0.0%	0.0%	0.0%	0.0%	100.0%	0.0%	0.0%	0.0%
CDC25B	0.0%	0.0%	0.0%	0.0%	100.0%	0.0%	0.0%	0.0%	0.0%
CDC42	0.0%	0.0%	0.0%	0.0%	100.0%	0.0%	0.0%	0.0%	0.0%
DAXX	0.0%	0.0%	0.0%	0.0%	100.0%	0.0%	0.0%	0.0%	0.0%
DUSP4	0.0%	0.0%	0.0%	0.0%	100.0%	0.0%	0.0%	0.0%	0.0%
EGFR	0.0%	0.0%	0.0%	0.0%	100.0%	0.0%	0.0%	0.0%	0.0%
FAS	0.0%	0.0%	0.0%	0.0%	0.0%	0.0%	0.0%	0.0%	100.0%
FGF13	0.0%	0.0%	0.0%	0.0%	0.0%	100.0%	0.0%	0.0%	0.0%
FGF19	25.0%	0.0%	0.0%	0.0%	75.0%	0.0%	0.0%	0.0%	0.0%

FGF3	0.0%	0.0%	0.0%	0.0%	100.0%	0.0%	0.0%	0.0%	0.0%
FGF4	0.0%	0.0%	0.0%	0.0%	100.0%	0.0%	0.0%	0.0%	0.0%
FGFR1	0.0%	0.0%	0.0%	0.0%	100.0%	0.0%	0.0%	0.0%	0.0%
FGFR2	0.0%	0.0%	0.0%	0.0%	100.0%	0.0%	0.0%	0.0%	0.0%
FGFR3	0.0%	28.6%	0.0%	0.0%	71.4%	0.0%	0.0%	0.0%	0.0%
FGFR4	0.0%	0.0%	0.0%	0.0%	100.0%	0.0%	0.0%	0.0%	0.0%
FLNB	0.0%	0.0%	0.0%	0.0%	100.0%	0.0%	0.0%	0.0%	0.0%
FLNC	0.0%	0.0%	0.0%	0.0%	100.0%	0.0%	0.0%	0.0%	0.0%
HRAS	0.0%	0.0%	0.0%	0.0%	100.0%	0.0%	0.0%	0.0%	0.0%
JUN	100.0%	0.0%	0.0%	0.0%	0.0%	0.0%	0.0%	0.0%	0.0%
KRAS	0.0%	0.0%	0.0%	0.0%	100.0%	0.0%	0.0%	0.0%	0.0%
MAP2K2	0.0%	0.0%	0.0%	0.0%	100.0%	0.0%	0.0%	0.0%	0.0%
MAP2K4	25.0%	0.0%	0.0%	0.0%	75.0%	0.0%	0.0%	0.0%	0.0%
MAP2K7	100.0%	0.0%	0.0%	0.0%	0.0%	0.0%	0.0%	0.0%	0.0%
MAP3K1	0.0%	33.3%	0.0%	0.0%	66.7%	0.0%	0.0%	0.0%	0.0%
MAP3K13	0.0%	0.0%	0.0%	0.0%	100.0%	0.0%	0.0%	0.0%	0.0%
MAP3K5	0.0%	0.0%	0.0%	0.0%	100.0%	0.0%	0.0%	0.0%	0.0%
MAP3K6	0.0%	0.0%	0.0%	0.0%	100.0%	0.0%	0.0%	0.0%	0.0%
MAP4K4	0.0%	0.0%	0.0%	0.0%	100.0%	0.0%	0.0%	0.0%	0.0%
MAPK3	0.0%	0.0%	0.0%	0.0%	100.0%	0.0%	0.0%	0.0%	0.0%
MAPK8IP3	0.0%	0.0%	0.0%	0.0%	100.0%	0.0%	0.0%	0.0%	0.0%
MOS	0.0%	0.0%	0.0%	0.0%	100.0%	0.0%	0.0%	0.0%	0.0%
MYC	0.0%	33.3%	0.0%	0.0%	66.7%	0.0%	0.0%	0.0%	0.0%
NF1	25.0%	0.0%	0.0%	0.0%	37.5%	0.0%	0.0%	37.5%	0.0%
NRAS	0.0%	0.0%	0.0%	0.0%	100.0%	0.0%	0.0%	0.0%	0.0%
NTRK1	28.6%	0.0%	0.0%	0.0%	71.4%	0.0%	0.0%	0.0%	0.0%
NTRK2	0.0%	0.0%	0.0%	0.0%	100.0%	0.0%	0.0%	0.0%	0.0%

PDGFRA	0.0%	0.0%	0.0%	0.0%	66.7%	33.3%	0.0%	0.0%	0.0%
PDGFRB	0.0%	0.0%	0.0%	0.0%	100.0%	0.0%	0.0%	0.0%	0.0%
PRKCG	0.0%	0.0%	0.0%	0.0%	100.0%	0.0%	0.0%	0.0%	0.0%
RAC2	0.0%	0.0%	0.0%	0.0%	100.0%	0.0%	0.0%	0.0%	0.0%
RAF1	33.3%	0.0%	0.0%	0.0%	66.7%	0.0%	0.0%	0.0%	0.0%
RASA1	27.3%	18.2%	9.1%	0.0%	27.3%	0.0%	0.0%	18.2%	0.0%
RPS6KA2	0.0%	0.0%	50.0%	0.0%	0.0%	50.0%	0.0%	0.0%	0.0%
RPS6KA4	11.1%	0.0%	0.0%	0.0%	88.9%	0.0%	0.0%	0.0%	0.0%
RRAS	0.0%	0.0%	0.0%	0.0%	100.0%	0.0%	0.0%	0.0%	0.0%
RRAS2	33.3%	0.0%	33.3%	0.0%	33.3%	0.0%	0.0%	0.0%	0.0%
SOS1	0.0%	0.0%	0.0%	0.0%	100.0%	0.0%	0.0%	0.0%	0.0%
TAOK2	0.0%	0.0%	0.0%	0.0%	100.0%	0.0%	0.0%	0.0%	0.0%
TGFBR1	16.7%	0.0%	16.7%	0.0%	66.7%	0.0%	0.0%	0.0%	0.0%
TGFBR2	46.2%	0.0%	0.0%	0.0%	53.8%	0.0%	0.0%	0.0%	0.0%
TP53	11.6%	3.9%	1.6%	1.6%	64.3%	11.6%	0.8%	4.7%	0.0%
TRAF2	0.0%	0.0%	0.0%	0.0%	100.0%	0.0%	0.0%	0.0%	0.0%

Table S6. Nature of the mutations of JAK/STAT pathway alterations in Late-Onset Colorectal Cancer (LOCRC) among Hispanic/Latino (H/L).

	Late-Onset Hispanic/Latino Samples				
	**Frame Shift Deletion**	**Frame Shift Insertion**	**In Frame Deletion**	**Missense Mutation**	**Nonsense Mutation**
**JAK1**	62.5%	25.0%	0.0%	12.5%	0.0%
**JAK2**	0.0%	0.0%	0.0%	50.0%	50.0%
**JAK3**	33.3%	0.0%	16.7%	50.0%	0.0%
**STAT3**	50.0%	0.0%	0.0%	50.0%	0.0%
**STAT5A**	16.7%	0.0%	16.7%	66.7%	0.0%
**STAT5B**	0.0%	66.7%	0.0%	33.3%	0.0%

Table S7. Nature of the mutations of MAPK pathway alterations in Early-Onset Colorectal Cancer (EOCRC) among Non-Hispanics Whites (NHW).

	Early-Onset NHW Samples								
	**Frame Shift Deletion**	**Frame Shift Insertion**	**In Frame Deletion**	**In Frame Insertion**	**Missense Mutation**	**Nonsense Mutation**	**Splice Region**	**Splice Site**	**Translation Start Site**
**ACVR1**	0.0%	8.3%	0.0%	0.0%	83.3%	8.3%	0.0%	0.0%	0.0%
**ACVR1B**	0.0%	0.0%	0.0%	0.0%	100.0%	0.0%	0.0%	0.0%	0.0%
**ACVR1C**	0.0%	0.0%	0.0%	0.0%	0.0%	100.0%	0.0%	0.0%	0.0%
**AKT1**	5.9%	0.0%	0.0%	0.0%	94.1%	0.0%	0.0%	0.0%	0.0%
**AKT2**	0.0%	0.0%	0.0%	0.0%	85.7%	14.3%	0.0%	0.0%	0.0%
**AKT3**	0.0%	0.0%	0.0%	0.0%	76.2%	23.8%	0.0%	0.0%	0.0%
**ARRB1**	0.0%	0.0%	0.0%	0.0%	0.0%	0.0%	100.0%	0.0%	0.0%
**ARRB2**	0.0%	0.0%	0.0%	0.0%	100.0%	0.0%	0.0%	0.0%	0.0%
**BDNF**	0.0%	0.0%	0.0%	0.0%	100.0%	0.0%	0.0%	0.0%	0.0%
**BRAF**	2.8%	2.8%	1.4%	0.0%	87.3%	2.8%	0.0%	2.8%	0.0%
**CACNA1A**	0.0%	0.0%	0.0%	0.0%	100.0%	0.0%	0.0%	0.0%	0.0%
**CACNA1B**	0.0%	0.0%	0.0%	0.0%	100.0%	0.0%	0.0%	0.0%	0.0%
**CACNA1C**	0.0%	0.0%	0.0%	0.0%	100.0%	0.0%	0.0%	0.0%	0.0%
**CACNA1D**	0.0%	0.0%	0.0%	0.0%	100.0%	0.0%	0.0%	0.0%	0.0%
**CACNA1E**	0.0%	0.0%	0.0%	0.0%	80.0%	20.0%	0.0%	0.0%	0.0%
**CACNA1F**	0.0%	0.0%	0.0%	0.0%	66.7%	33.3%	0.0%	0.0%	0.0%
**CACNA1G**	0.0%	0.0%	0.0%	0.0%	100.0%	0.0%	0.0%	0.0%	0.0%
**CACNA1H**	0.0%	0.0%	0.0%	0.0%	100.0%	0.0%	0.0%	0.0%	0.0%
**CACNA1I**	0.0%	0.0%	0.0%	0.0%	100.0%	0.0%	0.0%	0.0%	0.0%
**CACNA1S**	0.0%	0.0%	0.0%	0.0%	100.0%	0.0%	0.0%	0.0%	0.0%
**CACNA2D1**	0.0%	0.0%	0.0%	0.0%	100.0%	0.0%	0.0%	0.0%	0.0%

**CACNA2D3**	0.0%	33.3%	0.0%	0.0%	66.7%	0.0%	0.0%	0.0%	0.0%
**CACNA2D4**	0.0%	0.0%	0.0%	0.0%	100.0%	0.0%	0.0%	0.0%	0.0%
**CACNB1**	0.0%	0.0%	0.0%	0.0%	100.0%	0.0%	0.0%	0.0%	0.0%
**CACNB3**	0.0%	0.0%	0.0%	0.0%	100.0%	0.0%	0.0%	0.0%	0.0%
**CACNB4**	0.0%	0.0%	0.0%	0.0%	100.0%	0.0%	0.0%	0.0%	0.0%
**CACNG2**	0.0%	0.0%	0.0%	0.0%	100.0%	0.0%	0.0%	0.0%	0.0%
**CACNG3**	0.0%	0.0%	0.0%	0.0%	100.0%	0.0%	0.0%	0.0%	0.0%
**CACNG5**	0.0%	0.0%	0.0%	0.0%	100.0%	0.0%	0.0%	0.0%	0.0%
**CACNG6**	100.0%	0.0%	0.0%	0.0%	0.0%	0.0%	0.0%	0.0%	0.0%
**CDC25B**	50.0%	0.0%	0.0%	0.0%	50.0%	0.0%	0.0%	0.0%	0.0%
**CDC42**	50.0%	0.0%	0.0%	0.0%	50.0%	0.0%	0.0%	0.0%	0.0%
**CRKL**	14.3%	0.0%	0.0%	0.0%	85.7%	0.0%	0.0%	0.0%	0.0%
**DAXX**	6.3%	6.3%	0.0%	0.0%	87.5%	0.0%	0.0%	0.0%	0.0%
**DUSP3**	0.0%	0.0%	0.0%	0.0%	100.0%	0.0%	0.0%	0.0%	0.0%
**DUSP4**	33.3%	0.0%	0.0%	0.0%	66.7%	0.0%	0.0%	0.0%	0.0%
**DUSP7**	0.0%	0.0%	0.0%	0.0%	100.0%	0.0%	0.0%	0.0%	0.0%
**EGF**	0.0%	0.0%	0.0%	0.0%	100.0%	0.0%	0.0%	0.0%	0.0%
**EGFR**	0.0%	0.0%	0.0%	0.0%	100.0%	0.0%	0.0%	0.0%	0.0%
**ELK4**	50.0%	50.0%	0.0%	0.0%	0.0%	0.0%	0.0%	0.0%	0.0%
**FAS**	0.0%	50.0%	0.0%	0.0%	50.0%	0.0%	0.0%	0.0%	0.0%
**FGF10**	0.0%	0.0%	0.0%	0.0%	100.0%	0.0%	0.0%	0.0%	0.0%
**FGF11**	0.0%	0.0%	0.0%	0.0%	0.0%	0.0%	100.0%	0.0%	0.0%
**FGF13**	0.0%	0.0%	0.0%	0.0%	100.0%	0.0%	0.0%	0.0%	0.0%
**FGF17**	0.0%	0.0%	0.0%	0.0%	100.0%	0.0%	0.0%	0.0%	0.0%
**FGF19**	0.0%	0.0%	0.0%	0.0%	100.0%	0.0%	0.0%	0.0%	0.0%
**FGF23**	0.0%	0.0%	0.0%	0.0%	100.0%	0.0%	0.0%	0.0%	0.0%
**FGF3**	0.0%	0.0%	0.0%	0.0%	100.0%	0.0%	0.0%	0.0%	0.0%
**FGF4**	0.0%	0.0%	0.0%	0.0%	100.0%	0.0%	0.0%	0.0%	0.0%

**FGFR1**	4.3%	0.0%	0.0%	0.0%	91.3%	0.0%	0.0%	4.3%	0.0%
**FGFR2**	4.3%	0.0%	0.0%	0.0%	78.3%	17.4%	0.0%	0.0%	0.0%
**FGFR3**	7.7%	7.7%	0.0%	0.0%	80.8%	3.8%	0.0%	0.0%	0.0%
**FGFR4**	5.9%	0.0%	0.0%	0.0%	94.1%	0.0%	0.0%	0.0%	0.0%
**FLNB**	100.0%	0.0%	0.0%	0.0%	0.0%	0.0%	0.0%	0.0%	0.0%
**FLNC**	20.0%	0.0%	0.0%	0.0%	80.0%	0.0%	0.0%	0.0%	0.0%
**FOS**	100.0%	0.0%	0.0%	0.0%	0.0%	0.0%	0.0%	0.0%	0.0%
**GNG12**	100.0%	0.0%	0.0%	0.0%	0.0%	0.0%	0.0%	0.0%	0.0%
**HRAS**	0.0%	0.0%	0.0%	0.0%	100.0%	0.0%	0.0%	0.0%	0.0%
**IL1R1**	0.0%	0.0%	0.0%	0.0%	100.0%	0.0%	0.0%	0.0%	0.0%
**JUN**	28.6%	28.6%	0.0%	0.0%	35.7%	7.1%	0.0%	0.0%	0.0%
**KRAS**	0.0%	0.0%	0.0%	0.0%	99.7%	0.3%	0.0%	0.0%	0.0%
**MAP2K1**	6.3%	0.0%	0.0%	0.0%	93.8%	0.0%	0.0%	0.0%	0.0%
**MAP2K2**	0.0%	16.7%	0.0%	0.0%	83.3%	0.0%	0.0%	0.0%	0.0%
**MAP2K4**	3.8%	0.0%	3.8%	0.0%	46.2%	34.6%	0.0%	11.5%	0.0%
**MAP2K7**	0.0%	0.0%	0.0%	0.0%	100.0%	0.0%	0.0%	0.0%	0.0%
**MAP3K1**	9.4%	0.0%	3.1%	0.0%	62.5%	18.8%	0.0%	6.3%	0.0%
**MAP3K13**	13.0%	0.0%	8.7%	0.0%	56.5%	21.7%	0.0%	0.0%	0.0%
**MAP3K14**	0.0%	0.0%	0.0%	0.0%	0.0%	0.0%	0.0%	100.0%	0.0%
**MAP3K3**	0.0%	0.0%	0.0%	0.0%	100.0%	0.0%	0.0%	0.0%	0.0%
**MAP3K4**	100.0%	0.0%	0.0%	0.0%	0.0%	0.0%	0.0%	0.0%	0.0%
**MAP3K5**	0.0%	0.0%	0.0%	0.0%	100.0%	0.0%	0.0%	0.0%	0.0%
**MAP3K6**	0.0%	0.0%	0.0%	0.0%	100.0%	0.0%	0.0%	0.0%	0.0%
**MAP4K1**	0.0%	0.0%	0.0%	0.0%	100.0%	0.0%	0.0%	0.0%	0.0%
**MAP4K3**	0.0%	0.0%	0.0%	0.0%	100.0%	0.0%	0.0%	0.0%	0.0%
**MAP4K4**	0.0%	0.0%	0.0%	0.0%	100.0%	0.0%	0.0%	0.0%	0.0%
**MAPK1**	0.0%	0.0%	14.3%	0.0%	85.7%	0.0%	0.0%	0.0%	0.0%
**MAPK10**	0.0%	0.0%	0.0%	0.0%	100.0%	0.0%	0.0%	0.0%	0.0%

**MAPK13**	0.0%	0.0%	0.0%	0.0%	100.0%	0.0%	0.0%	0.0%	0.0%
**MAPK14**	0.0%	0.0%	0.0%	0.0%	100.0%	0.0%	0.0%	0.0%	0.0%
**MAPK3**	0.0%	0.0%	0.0%	0.0%	85.7%	14.3%	0.0%	0.0%	0.0%
**MAPK8**	0.0%	0.0%	0.0%	0.0%	50.0%	50.0%	0.0%	0.0%	0.0%
**MAPK8IP1**	0.0%	0.0%	0.0%	0.0%	100.0%	0.0%	0.0%	0.0%	0.0%
**MAPK8IP2**	0.0%	0.0%	0.0%	0.0%	100.0%	0.0%	0.0%	0.0%	0.0%
**MAPK8IP3**	0.0%	0.0%	0.0%	0.0%	100.0%	0.0%	0.0%	0.0%	0.0%
**MAPT**	0.0%	0.0%	0.0%	0.0%	100.0%	0.0%	0.0%	0.0%	0.0%
**MAX**	0.0%	0.0%	0.0%	0.0%	83.3%	16.7%	0.0%	0.0%	0.0%
**MOS**	0.0%	0.0%	0.0%	0.0%	100.0%	0.0%	0.0%	0.0%	0.0%
**MYC**	0.0%	0.0%	16.7%	0.0%	83.3%	0.0%	0.0%	0.0%	0.0%
**NF1**	7.9%	13.2%	0.0%	0.0%	60.5%	13.2%	0.0%	5.3%	0.0%
**NFATC2**	0.0%	0.0%	0.0%	0.0%	100.0%	0.0%	0.0%	0.0%	0.0%
**NFATC4**	0.0%	0.0%	0.0%	0.0%	100.0%	0.0%	0.0%	0.0%	0.0%
**NFKB1**	0.0%	0.0%	0.0%	0.0%	100.0%	0.0%	0.0%	0.0%	0.0%
**NRAS**	0.0%	0.0%	0.0%	0.0%	100.0%	0.0%	0.0%	0.0%	0.0%
**NTRK1**	4.2%	4.2%	0.0%	0.0%	91.7%	0.0%	0.0%	0.0%	0.0%
**NTRK2**	0.0%	5.0%	0.0%	0.0%	90.0%	5.0%	0.0%	0.0%	0.0%
**PAK1**	0.0%	8.3%	0.0%	0.0%	83.3%	0.0%	0.0%	8.3%	0.0%
**PAK2**	0.0%	0.0%	0.0%	0.0%	100.0%	0.0%	0.0%	0.0%	0.0%
**PDGFB**	0.0%	0.0%	0.0%	0.0%	100.0%	0.0%	0.0%	0.0%	0.0%
**PDGFRA**	2.0%	0.0%	0.0%	0.0%	81.6%	10.2%	0.0%	6.1%	0.0%
**PDGFRB**	0.0%	0.0%	0.0%	0.0%	95.5%	0.0%	0.0%	4.5%	0.0%
**PPM1B**	0.0%	0.0%	0.0%	0.0%	100.0%	0.0%	0.0%	0.0%	0.0%
**PPP3CB**	0.0%	0.0%	0.0%	0.0%	100.0%	0.0%	0.0%	0.0%	0.0%
**PPP3R2**	0.0%	0.0%	0.0%	0.0%	0.0%	100.0%	0.0%	0.0%	0.0%
**PPP5C**	0.0%	0.0%	0.0%	0.0%	100.0%	0.0%	0.0%	0.0%	0.0%
**PRKACG**	0.0%	0.0%	0.0%	0.0%	100.0%	0.0%	0.0%	0.0%	0.0%

**PRKCG**	0.0%	0.0%	0.0%	0.0%	100.0%	0.0%	0.0%	0.0%	0.0%
**PTPN5**	0.0%	100.0%	0.0%	0.0%	0.0%	0.0%	0.0%	0.0%	0.0%
**PTPN7**	0.0%	0.0%	0.0%	0.0%	100.0%	0.0%	0.0%	0.0%	0.0%
**PTPRR**	0.0%	0.0%	0.0%	0.0%	100.0%	0.0%	0.0%	0.0%	0.0%
**RAC1**	12.5%	0.0%	0.0%	0.0%	87.5%	0.0%	0.0%	0.0%	0.0%
**RAC2**	0.0%	0.0%	0.0%	0.0%	100.0%	0.0%	0.0%	0.0%	0.0%
**RAC3**	0.0%	0.0%	0.0%	0.0%	100.0%	0.0%	0.0%	0.0%	0.0%
**RAF1**	0.0%	0.0%	0.0%	0.0%	91.7%	8.3%	0.0%	0.0%	0.0%
**RAP1A**	0.0%	0.0%	0.0%	0.0%	100.0%	0.0%	0.0%	0.0%	0.0%
**RAPGEF2**	0.0%	0.0%	0.0%	0.0%	100.0%	0.0%	0.0%	0.0%	0.0%
**RASA1**	10.6%	2.1%	0.0%	0.0%	68.1%	14.9%	0.0%	4.3%	0.0%
**RASA2**	0.0%	0.0%	0.0%	0.0%	100.0%	0.0%	0.0%	0.0%	0.0%
**RASGRF1**	0.0%	0.0%	0.0%	0.0%	100.0%	0.0%	0.0%	0.0%	0.0%
**RASGRF2**	0.0%	0.0%	0.0%	0.0%	100.0%	0.0%	0.0%	0.0%	0.0%
**RASGRP1**	0.0%	0.0%	0.0%	0.0%	100.0%	0.0%	0.0%	0.0%	0.0%
**RASGRP2**	0.0%	0.0%	0.0%	0.0%	100.0%	0.0%	0.0%	0.0%	0.0%
**RASGRP3**	0.0%	0.0%	0.0%	0.0%	100.0%	0.0%	0.0%	0.0%	0.0%
**RPS6KA1**	0.0%	0.0%	0.0%	0.0%	100.0%	0.0%	0.0%	0.0%	0.0%
**RPS6KA2**	0.0%	0.0%	0.0%	0.0%	100.0%	0.0%	0.0%	0.0%	0.0%
**RPS6KA3**	0.0%	0.0%	0.0%	0.0%	100.0%	0.0%	0.0%	0.0%	0.0%
**RPS6KA4**	30.0%	0.0%	0.0%	0.0%	65.0%	0.0%	0.0%	0.0%	5.0%
**RPS6KA6**	0.0%	0.0%	0.0%	0.0%	100.0%	0.0%	0.0%	0.0%	0.0%
**RRAS**	0.0%	0.0%	0.0%	0.0%	100.0%	0.0%	0.0%	0.0%	0.0%
**RRAS2**	0.0%	0.0%	0.0%	0.0%	100.0%	0.0%	0.0%	0.0%	0.0%
**SOS1**	8.6%	2.9%	0.0%	0.0%	74.3%	14.3%	0.0%	0.0%	0.0%
**TAOK1**	0.0%	0.0%	0.0%	0.0%	0.0%	100.0%	0.0%	0.0%	0.0%
**TAOK2**	0.0%	0.0%	0.0%	0.0%	100.0%	0.0%	0.0%	0.0%	0.0%
**TAOK3**	0.0%	50.0%	0.0%	0.0%	50.0%	0.0%	0.0%	0.0%	0.0%

**TGFB2**	0.0%	0.0%	0.0%	0.0%	66.7%	33.3%	0.0%	0.0%	0.0%
**TGFB3**	100.0%	0.0%	0.0%	0.0%	0.0%	0.0%	0.0%	0.0%	0.0%
**TGFBR1**	3.8%	0.0%	0.0%	0.0%	65.4%	26.9%	0.0%	3.8%	0.0%
**TGFBR2**	33.3%	2.4%	7.1%	2.4%	52.4%	0.0%	0.0%	0.0%	2.4%
**TP53**	7.5%	3.7%	1.2%	0.7%	64.1%	16.1%	0.1%	6.5%	0.0%
**TRAF2**	12.5%	6.3%	0.0%	0.0%	62.5%	18.8%	0.0%	0.0%	0.0%

Table S8. Nature of the mutations of JAK/STAT pathway alterations in Early-Onset Colorectal Cancer (EOCRC) among Non-Hispanics Whites (NHW).

	Early-Onset NHW Samples					
	**Frame Shift Deletion**	**Frame Shift Insertion**	**In Frame Deletion**	**Missense Mutation**	**Nonsense Mutation**	**Splice Site**
JAK1	29.0%	6.5%	0.0%	61.3%	0.0%	3.2%
JAK2	4.3%	8.7%	0.0%	73.9%	13.0%	0.0%
JAK3	15.4%	0.0%	0.0%	76.9%	0.0%	7.7%
PIAS1	0.0%	0.0%	0.0%	100.0%	0.0%	0.0%
PIAS2	0.0%	0.0%	0.0%	0.0%	100.0%	0.0%
PTPRC	33.3%	0.0%	0.0%	66.7%	0.0%	0.0%
SOCS1	0.0%	33.3%	0.0%	66.7%	0.0%	0.0%
STAT1	0.0%	0.0%	0.0%	100.0%	0.0%	0.0%
STAT3	6.3%	6.3%	6.3%	56.3%	18.8%	6.3%
STAT5A	7.7%	7.7%	7.7%	69.2%	7.7%	0.0%
STAT5B	47.1%	0.0%	0.0%	47.1%	5.9%	0.0%

Supplemental Figures

Figure S1. Overall survival curves of early-onset Non-Hispanic White (NHW) patients stratified by the presence or absence of MAPK (left) and JAK/STAT (right) pathway alterations. The left panel displays the Kaplan-Meier survival curve for early-onset colorectal cancer (EOCRC) NHW patients stratified by MAPK pathway alterations. Patients with MAPK pathway alterations (red curve) did not exhibit a significant difference in overall survival compared to those without alterations (blue curve), with a p-value of 0.49. The shaded regions represent 95% confidence intervals, and vertical tick marks indicate censored patients. The right panel presents the Kaplan-Meier survival curve for early-onset NHW CRC patients stratified by JAK/STAT pathway alterations. Patients with JAK/STAT pathway alterations (red curve) showed a significant difference in survival outcomes compared to those without alterations (blue curve), with a p-value < 0.0001, suggesting a strong prognostic impact of JAK/STAT pathway disruptions in this population.

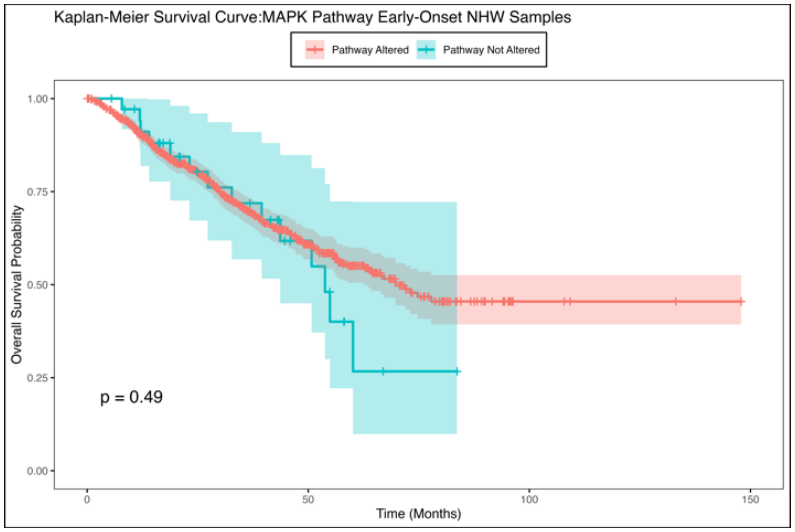

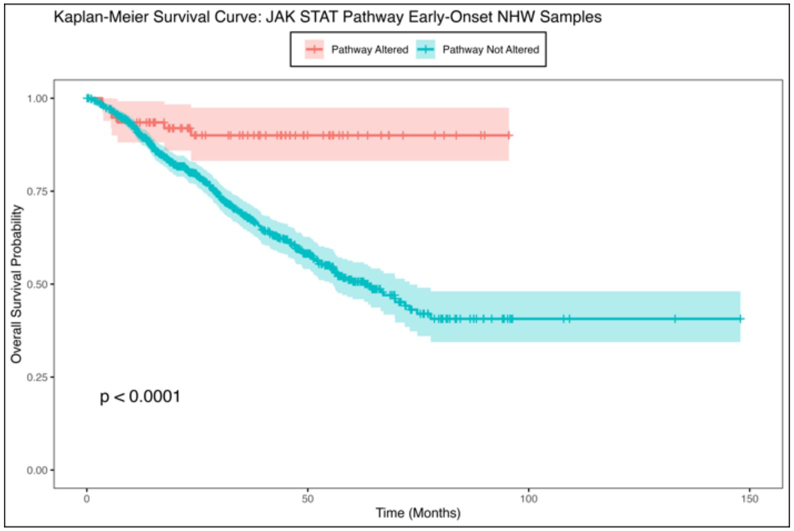

Supplement: Supplementary file 1 [file cancers-17-01093-s001.zip › cancers-3511944-supplementary.pdf]
